# Relationships Between Bronchodilators, Steroids, Antiarrhythmic Drugs, Antidepressants, and Benzodiazepines and Heart Disease and Ischemic Stroke in Patients With Predominant Bronchiectasis and Asthma

**DOI:** 10.3389/fcvm.2022.797623

**Published:** 2022-02-17

**Authors:** Jun-Jun Yeh, Mei-Chu Lai, Yu-Cih Yang, Chung-Y. Hsu, Chia-Hung Kao

**Affiliations:** ^1^Department of Family Medicine, Chest Medicine, Geriatric Medicine and Medical Research, Ditmanson Medical Foundation Chia-Yi Christian Hospital, Chiayi, Taiwan; ^2^College of Medicine, China Medical University, Taichung, Taiwan; ^3^Department of Laboratory Medicine, Ditmanson Medical Foundation Chia-Yi Christian Hospital, Chiayi, Taiwan; ^4^Management Office for Health Data, China Medical University Hospital, Taichung, Taiwan; ^5^Graduate Institute of Biomedical Sciences, College of Medicine, China Medical University, Taichung, Taiwan; ^6^Center of Augmented Intelligence in Healthcare, China Medical University Hospital, Taichung, Taiwan; ^7^Department of Nuclear Medicine and PET Center, China Medical University Hospital, Taichung, Taiwan; ^8^Department of Bioinformatics and Medical Engineering, Asia University, Taichung, Taiwan

**Keywords:** heart disease, ischemic stroke, bronchiectasis-asthma combination, NHIRD, National Health Insurance Research Database, medicine

## Abstract

**Objective:**

We investigated the effects of medication on heart disease and ischemic stroke (HDS) risk in patients with predominant bronchiectasis-asthma combination (BCAS).

**Methods:**

BCAS and non-BCAS cohorts (*N* = 588 and 1,118, respectively) were retrospectively enrolled. The cumulative incidence of HDS was analyzed using Cox proportional regression; propensity scores were estimated using non-parsimonious multivariable logistic regression. Adjusted hazard ratios (aHRs) and 95% confidence intervals (CIs) for HDS were calculated, adjusting for sex, age, comorbidities, and medication {long- and short-acting β2 agonists and muscarinic antagonists (LABAs/SABAs and LAMAs/SAMAs), steroids [inhaled corticosteroid steroids (ICSs), oral steroids (OSs)], antiarrhythmics, antidepressants (fluoxetine), benzodiazepines (alprazolam, fludiazepam), statins and antihypertensive drugs (diuretics, cardioselective beta blockers, calcium channel blockers (CCBs) and angiotensin converting enzyme inhibitors (ACEi), angiotensin II blockers)}.

**Results:**

Compared with the non-BCAS cohort, the BCAS cohort taking LABAs, SABAs, SAMAs, ICSs, OSs, antiarrhythmics, and alprazolam had an elevated HDS risk [aHRs (95% CIs): 2.36 (1.25–4.33), 2.65 (1.87–3.75), 2.66 (1.74–4.05), 2.53 (1.61–3.99), 1.76 (1.43–2.18), 9.88 (3.27–30.5), and 1.73 (1.15–2.58), respectively except fludiazepam 1.33 (0.73–2.40)]. The aHRs (95% CIs) for LABAs ≤ 30 days, DDDs <415, ICSs ≤ 30 days were 1.10 (0.38–3.15), 2.95 (0.22–38.8), 1.45 (0.76–2.77). The aHRs (95% CIs) for current and recent alprazolam were 1.78 (1.09–2.93) and 777.8 (1.34–451590.0); for current and past fludiazepam were 1.39 (0.75–2.59) and 1.29 (0.42–4.01) and for past alprazolam was 1.57 (0.55–4.46); respectively. The aHRs (95% CIs) for alprazolam >30 DDDs, fludiazepam >20 DDDs, ICSs ≦415 DDDs, and OSs DDDs ≦15 were 1.60 (0.78–3.29), 2.43 (0.90–6.55), 5.02 (1.76–14.3), and 2.28 (1.43–3.62), respectively.

**Conclusion:**

The bronchodilators, steroids, and antiarrhythmics were associated with higher risk of HDS, even low dose use of steroids. However, the current use of LABAs/ICSs were not associated with HDS. Benzodiazepines were relatively safe, except for current or recent alprazolam use. Notably, taking confounders into account is crucial in observational studies.

## Introduction

Asthma and bronchiectasis are chronic inflammatory diseases (1–4). Bronchiectasis may be linked to asthma (BCAS) and is a frequent comorbidity (3, 5–7). BCAS is associated with frequent hospitalization, and a high blood eosinophil count is an additional phenotypic feature of severe eosinophilic asthma. To ensure precise and personalized treatment, BCAS should be considered as a separate entity (3, 5–7).

In the era of COVID-19, heart disease and ischemic stroke (HDS) has been reported as the most severe complication in patients with BCAS (8). Moreover, BCAS is associated with diseases related to arterial thrombosis, such as myocardial infarction and ischemic stroke (9). Psychiatric problems have also been observed in patients with COVID-19 and BCAS (10). Therefore, the effect of medications such as antianxiety drugs [benzodiazepines (BZDs)] in patients with BCAS is an urgent Research Topic.

We speculated that the high level of inflammation associated with atherosclerosis increases the risk of HDS (11, 12). Thus, we investigated the relationship between HDS and various drugs, including bronchodilators, steroids, antiarrhythmic drugs, anti-depressants, BZDs, and antihypertensive drugs in patients with BCAS cohort from the general population.

## Methods

### Data Source

To clarify the risk of HDS in the BCAS cohort, we used the Longitudinal Health Insurance Database 2000 (LHID 2000) compiled by the Taiwan National Health Research Institutes. International Classification of Diseases, Ninth Revision, Clinical Modification (ICD-9-CM) diagnoses (maximum of five) were recorded in this study. In the National Health Insurance Research Database (NHIRD), ICD-9-CM codes and the ICD-9 Procedure Coding System (ICD-9-PCS) were adopted to define diagnostic and procedure codes, respectively. Pursuant to the Personal Information Protection Act, individual identifiers are encrypted before being released for research. The NHIRD has been used in various studies and provides high-quality information on diagnoses, hospitalizations, and prescriptions.

### Ethics Statement

The NHIRD encrypts personal information to protect patients' privacy. It provides researchers with anonymous identification numbers associated with relevant claims information, including sex, date of birth, medical services received, and prescriptions. Therefore, patient consent is not required to access the NHIRD. The study protocol was approved by the Institutional Review Board of China Medical University (CMUH104-REC2-115-AR4), which also specifically waived the informed consent requirement.

### Study Population

**Figure 1:**
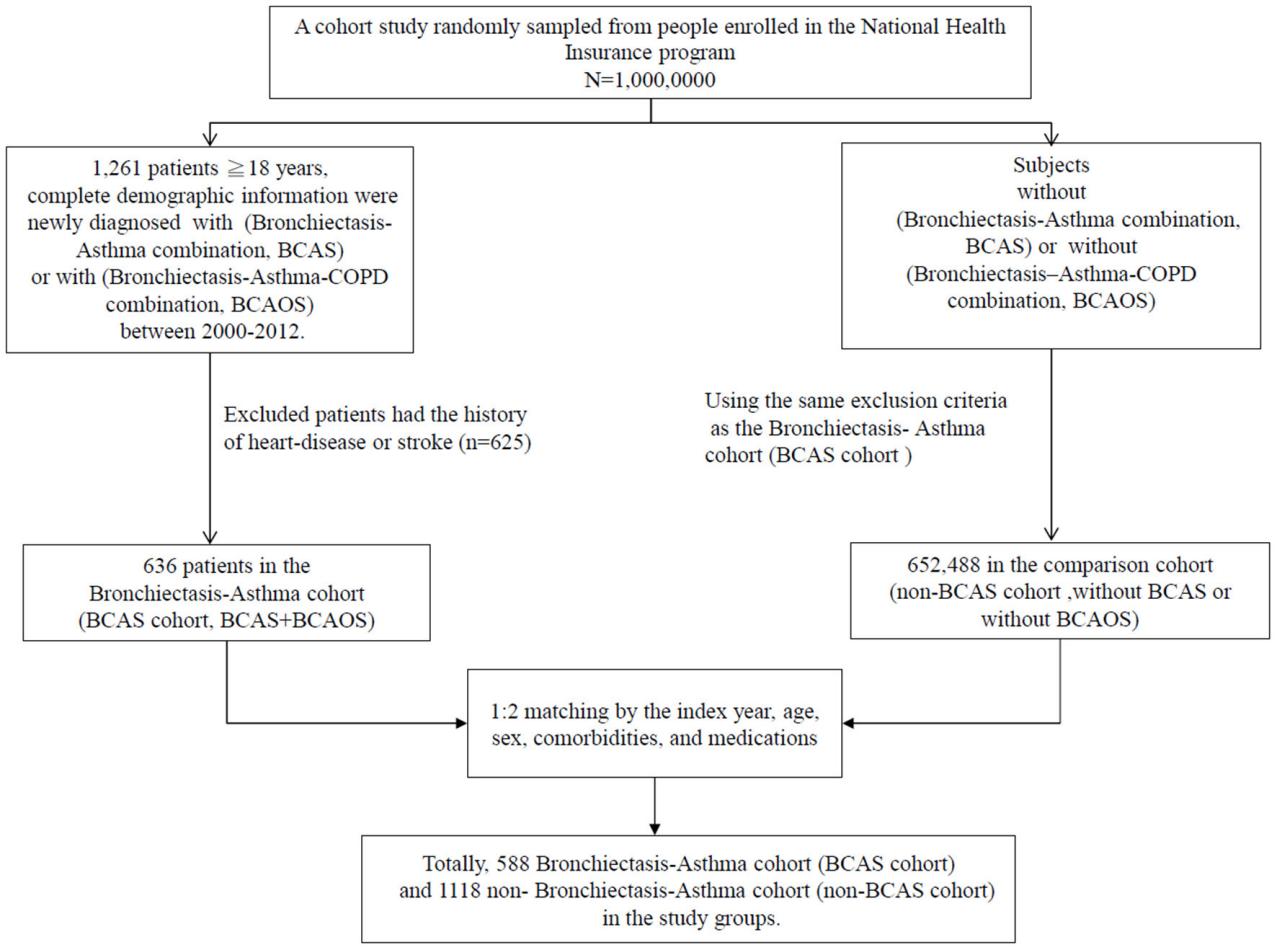
Flow chart of the selection of patients.

**Figure 2:**
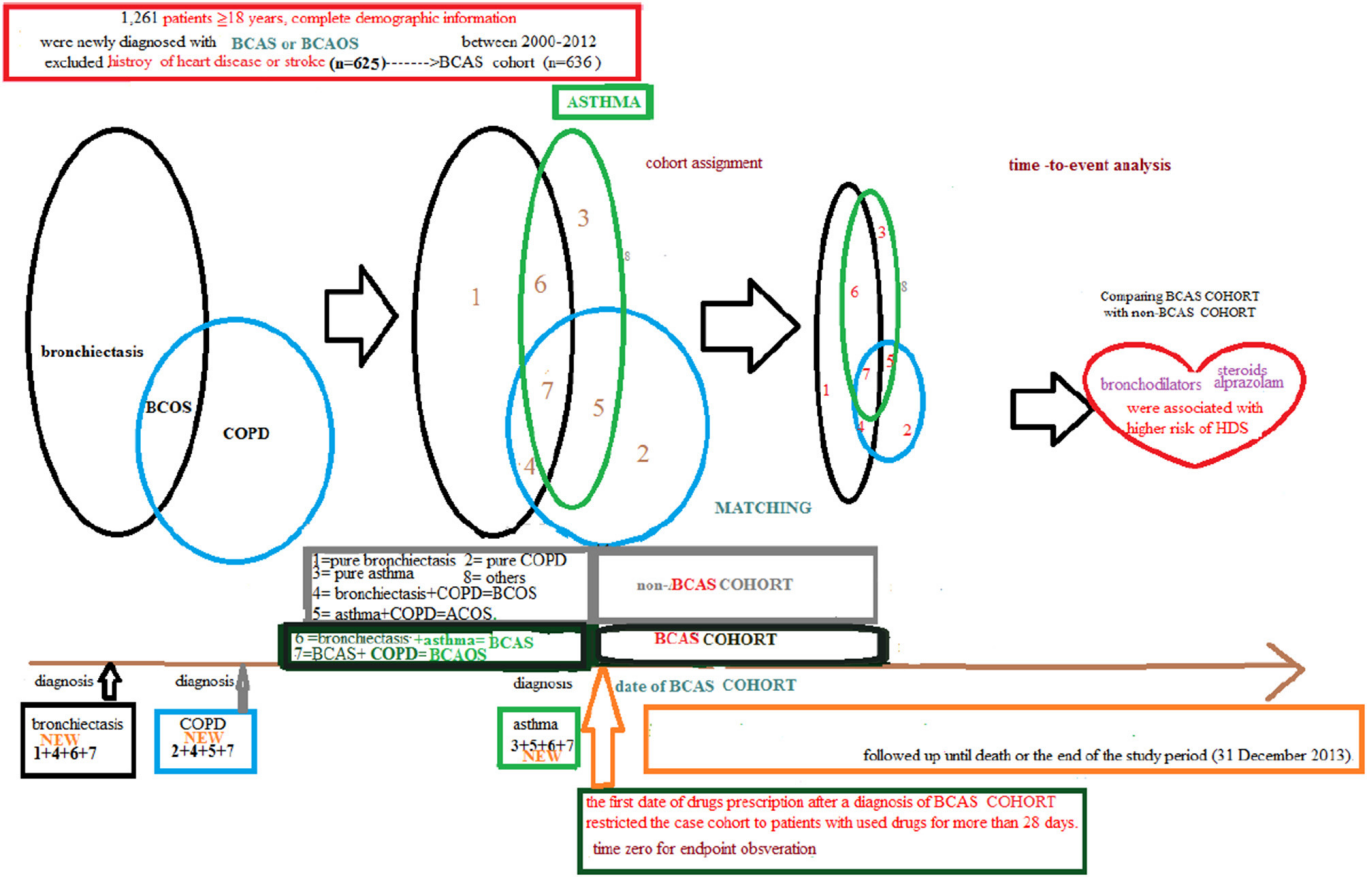
Full name of the subgroups of cohort with bronchiectasis-asthma combination cohort and cohort without the bronchiectasis–asthma combination.

This BCAS cohort was selected from the cumulative outpatient and inpatient population from the LHID 2000. Figures 1, 2 shows the process of selecting participants for study cohorts. We identified patients diagnosed with new bronchiectasis (ICD-9-CM code 494) or with new chronic obstructive pulmonary disease (COPD, ICD-9-CM Codes 491, 492, and 496) from claims data for 2000–2012.

The primary exclusion criteria were: (1) aged <18 years; (2) incomplete demographic information. The inclusion criteria (*n* = 1,261) were new diagnoses of asthma, bronchiectasis, and COPD having two outpatients visits or one inpatient visit. Patients aged ≥18 years having (the new bronchiectasis and new asthma combination [(ICD-9-CM Code 493), BCAS] or new BCAS and new COPD combination (BCAOS) were selected for the BCAS cohort entered into study. The control groups (non-BCAS cohort) were selected from the population without BCAS cohort. The non-BCAS cohort including the rest of the bronchiectasis or COPD or asthma or patients with immunosuppressants such as steroids use who are without a diagnosis of BCAS cohort. The secondary exclusion criteria including: diagnoses of heart disease or stroke (*n* = 625) before entry into the study. Before matching, ACOS cohort included 636 patients, non-BCAS cohort included 652,488 subjects. The study period was from January 1, 2000 to December 31, 2013 (Figures 1, 2).

Patients in the BCAS cohort were matched to individuals in the non-BCAS cohort according to gender, age (5-year span), comorbidities, medications, and year of entry into the study by frequency matching. After 1: 2 matching, the BCAS (*n* = 588) including the (6, pure BCAS and 7, BCAS+COPD, BCAOS). The non-BCAS cohort (*n* = 1,118) including the (1: pure bronchiectasis) = ((1 + 4 + 6 + 7, new bronchiectasis) – (4, BCOS) – (6, BCAS) – (7, BCAOS)), (2: pure COPD) = ((2 + 4 + 5 + 7, new COPD) – (4, BCOS) – (5, ACOS) – (7, BCAOS)), (3: pure asthma) = ((3 + 5 + 6 + 7, new asthma) – (5, ACOS) – (6, BCAS) – (7, BCAOS)), (4: bronchiectasis + COPD, BCOS), (5: asthma + COPD, ACOS) and (8: others – such as patients with steroids use) (Figure 2). We defined the index date of case-cohort by the first date of drugs prescription after a diagnosis of BCAS and we restricted the case-cohort to patients with used drugs for more than 28 days. [For the ICD-9-CM codes for comorbidities and the Anatomical Therapeutic Chemical (ATC) codes for medications, see Supplementary Table 1].

These patients were followed up until the occurrence of heart disease (ICD-9-CM codes 410–414, 425–429) or ischemic stroke (ICD-9-CM codes 433, 434, 435, and 436), death, withdrawal from the insurance program, or the end of the study period (December 31, 2013). For full names of comorbidities and medications (Supplementary Table 1).

### Statistical Analysis

The propensity scores (PS) for each patient were estimated using non-parsimonious multivariable logistic regression, with receipt of patients with or without BCAS cohort as the independent variable. We incorporated clinically relevant covariates (comorbidities, drugs, etc.) into our analysis—the primary analysis. The (heart disease or ischemic stroke, HDS) as dependent variables (13).

The BCAS cohort was compared with the non-BCAS cohort concerning variables, and the Wilcoxon rank-sum test was used to compare continuous variables between the BCAS cohort and the non-BCAS cohort, as necessary. The incidence density rates (per 1,000 person-years) were analyzed to estimate the HDS incidence in the BCAS cohort and the non-BCAS cohort stratified by gender, age, comorbidities, and medications. The annual incidence density rate was calculated by dividing the number of newly diagnosed HDS cases by the number of person-years at risk for BCAS cohort in each subcohort from 2000 to 2013. The comparison of the risk of HDS between the BCAS cohort and the non-BCAS cohort was calculated using Cox proportional hazard regression models. The analysis was adjusted for gender, age, comorbidities, and medications. The significance threshold was set at α = 0.05 for the a priori hypotheses. All analyses were performed using SAS statistical software (Version 9.4 for Windows; SAS Institute, Inc., Cary, NC, USA).

## Results

### Baseline Characteristics of the Study Population of the Propensity Score-Matched Population

Table 1 displays the distributions of age, comorbidities, and medications between the two cohorts. After PS-matching, the BCAS cohort comprised 588 patients, and the non-BCAS cohort included 1,118 patients. The two cohorts had a similar gender distribution. The mean age (SD) of patients was 54.66 (±32.2) years in the BCAS cohort and 56.53 (±34.0) years in the non-BCAS cohort (Wilcoxon rank-sum test, *p* = 0.02). Patients were predominately aged between 40 and 64 years. The demographic data of the BCAS cohort were similar to those of the non-BCAS cohort in terms of gender, age, comorbidities grouped and medications (bronchodilators, steroids, antiarrhythmic drugs, antidepressants, BZDs, statins, and antihypertensive drugs), with no significant differences between the BCAS cohort and non-BCAS cohort, except the use of long-acting β2 agonists (LABAs), inhaled corticosteroid steroids (ICSs), diuretics, cardioselective beta blockers, angiotensin converting enzyme inhibitors (ACEi), and calcium channel blockers (CCBs) were significantly more frequent in the BCAS cohort than in the non-BCAS cohort.

**Table 1:** Baseline characteristics of study population before and after matching based on propensity scores between two cohorts.

**Variable**	**Original population**				*p*-value^*^	**PS-matching population**				*p*-value^*^
	**BCAS cohort (*****n*** **=** **636)**		**Non-BCAS cohort (*****n*** **=** **652,488)**			**BCAS cohort (*****n*** **=** **588)**		**Non-BCAS cohort (*****n*** **=** **1,118)**		
	***N***	**%**	***N***	**%**		***N***	**%**	***N***	**%**	
Gender					0.0014					0.11
Female	350	55.0	317,605	48.7		323	54.9	569	60.9	
Male	286	45.0	334,883	51.3		265	45.1	549	49.1	
Age at baseline, year					<0.0001					0.001
<20	27	4.25	146,757	22.4		25	4.25	54	4.83	
20–39	87	13.6	267,629	41.0		81	13.7	153	13.6	
40–64	333	52.3	207,251	31.7		315	53.5	494	44.1	
≥65	189	29.7	30,855	4.73		167	28.4	417	37.3	
Mean (SD)^†^	54.92 (32.3)		34.35 (49.6)		<0.0001	54.66 (32.2)		56.53 (34.0)		0.02
Comorbidity										
Pulmonary tuberculosis	78	12.2	2,428	0.37	<0.0001	70	11.9	116	10.3	0.33
Non-tuberculosis mycobacterium	5	0.79	148	0.02	<0.0001	4	0.68	6	0.54	0.71
Rheumatoid arthritis	13	2.04	5,043	0.77	0.0003	9	1.53	22	1.97	0.52
Diffuse connective disease	12	1.89	4,599	0.70	0.0004	10	1.70	24	2.15	0.53
Pneumonia	202	31.7	22,265	3.41	<0.0001	177	30.1	329	29.4	0.77
COPD	349	54.8	18,575	2.85	<0.0001	315	53.5	632	56.5	0.24
Diabetes	69	10.8	21,527	3.30	<0.0001	64	10.8	128	11.4	0.72
Aspergillosis	2	0.31	20	0.003	<0.0001	2	0.34	0	0	0.05
Candiasis	1	0.16	14	0.002	<0.0001	1	0.17	1	0.09	0.64
Endemic mycoses	0	0	41	0.01	0.84	0	0	0	0	–
Mounier-Kuhn	0	0	59	0.01	0.81	0	0	0	0	–
Cystic fibrosis	0	0	3	0.0004	0.95	0	0	0	0	–
Hypertension	216	33.9	56,003	8.57	<0.0001	194	32.9	405	36.2	0.18
Hyperlipidemia	100	15.7	38,046	5.83	<0.0001	93	15.8	218	19.5	0.06
Pulmonary embolism	0	0	84	0.01	0.77	0	0	2	0.18	0.30
Depression	5	0.79	3,056	0.47	0.24	5	0.85	10	0.89	0.92
Smoking										
Tobacco dependence	1	0.16	597	0.09	0.58	1	0.17	2	0.18	0.96
Tobacco use disorder complicating pregnancy	0	0	0	0	–	0	0	0	0	–
Medication										
LABA	132	20.7	582	0.09	<0.0001	116	19.73	149	13.3	0.0005
LAMA	13	2.04	95	0.01	<0.0001	12	2.04	16	1.43	0.34
SABA	260	40.8	13,426	2.06	<0.0001	234	39.8	432	38.6	0.64
SAMA	179	28.1	8,524	1.31	<0.0001	159	27.0	299	26.7	0.89
ICSs	209	32.8	937	0.14	<0.0001	184	31.2	239	21.3	<0.0001
Oss	585	91.9	469,554	71.96	<0.0001	538	91.5	1028	91.9	0.74
Anti- arrhythmic	46	7.23	15,413	2.36	<0.0001	43	7.31	81	7.25	0.95
Alprazolam	169	26.5	71,361	10.9	<0.0001	155	26.3	296	26.4	0.95
Fluoxetine	0	0	224	0.03	0.64	0	0	0	0	–
Fludiazepam	80	12.5	28,789	4.41	<0.0001	72	12.2	144	12.8	0.70
Statins	52	8.18	29,369	4.50	<0.0001	52	8.84	125	11.2	0.13
Anti-hypertensive drugs										
Diuretics	73	11.5	31,369	4.81	<0.0001	70	11.9	261	23.4	<0.0001
Beta blockers	84	13.2	64,661	9.91	0.005	82	14.0	199	17.8	0.04
Calcium channel blockers	128	20.1	59,686	9.15	<0.0001	125	21.3	313	28.0	0.003
Angiotensin converting enzyme inhibitors	43	6.76	24,049	3.69	<0.0001	42	7.14	123	11.0	0.01
Angiotensin II blockers	53	8.33	13,320	2.04	<0.0001	49	8.33	96	8.59	0.86

* *P-value using chi-square for the comparisons between with and without BCAS cohort*.

*Average age using Wilcoxon rank-sum test for verification*.

*BCAS cohort, Bronchiectasis-Asthma combination cohort; COPD, Chronic obstructive pulmonary disease; LABAs/LAMAs, long-acting β2-agonist or muscarinic antagonist; SABAs/SAMAs, short-acting β2-agonist or muscarinic antagonist, steroids; ICSs, inhaled corticosteroid steroids; Oss, oral steroids; Beta blockers, cardioselective beta blockers (atenol, bisoprolol, metoprolol)*.

### Comparison of HDS Risk Between the BCAS Cohort and Non-BCAS Cohorts, With Patients Without Comorbidities or Medications as the Reference Group

As shown in Table 2, the incidence density rates of HDS were higher in the BCAS cohort than in the non-BCAS cohort (51.5 vs. 33.1 per 1,000 person-years). The results revealed that BCAS cohort had a higher risk of HDS than the non-BCAS cohort [adjusted hazard ratio (aHR) = 1.79; 95% confidence interval (CI) = 1.48–2.18]. The risks of HDS were 13.5-fold and 23.5-fold higher in patients aged 40–64 years (95% CI = 3.33–54.7) and ≥65 years (95% CI = 5.74–96.0), the patients aged <20 years as reference. Patients with rheumatoid arthritis (adjusted HR = 2.47; 95% CI = 1.41–4.32), diabetes (aHR = 1.35; 95% CI = 1.04–1.76), and hypertension (aHR = 1.67; 95% CI = 1.35–2.07) had a significantly elevated risk of HDS, patients without comorbidities as reference. Patients taking SABAs (aHR = 0.67; 95% CI = 0.54–0.83), OSs (aHR = 0.31; 95% CI = 0.23–0.41), statins (aHR = 0.50; 95 % = 0.35–0.71), and CCBs (aHR = 0.67; 95 % = 0.53–0.85) had a significantly lower risk of HDS, with patients not using the (SABAs, OSs, statins, CCBs) as references.

**Table 2:** Cox model measured hazard ratios and 95% confidence interval of heart-disease or ischemic stroke associated with gender, age, and comorbidity after propensity matching between two cohorts.

	**Heart-disease or ischemic stroke**			**Crude HR (95%CI)**	**Adjusted (95%CI)**
	**Event**	**PY**	**IR**		
BCAS cohort					
No	250	7,549	33.1	1 (reference)	1 (reference)
Yes	182	3,532	51.5	1.54 (1.28–1.87)^***^	1.79 (1.48–2.18)^***^
Gender					
Female	213	6,060	35.1	1 (reference)	1 (reference)
Male	219	5,021	43.6	1.22 (1.01–1.47)^*^	1.19 (0.98–1.45)
Age					
<20	2	861	2.32	1 (reference)	1 (reference)
20–39	16	2,090	7.65	3.25 (0.74–14.15)	2.54 (0.58–11.1)
40–64	203	5,270	38.5	15.94 (3.95–64.19)^***^	13.5 (3.33–54.7)^***^
≥65	211	2,860	73.7	29.9 (7.42–120.48)^***^	23.5 (5.74–96.0)^***^
Comorbidity					
Pulmonary tuberculosis					
No	388	10,132	38.2	1 (reference)	1 (reference)
Yes	44	949	46.3	1.16 (0.85–1.58)	–
Non-tuberculosis mycobacterium					
No	430	11,036	38.9	1 (reference)	1 (reference)
Yes	2	45	44.4	1.07 (0.26–4.32)	–
Rheumatoid arthritis					
No	419	10,939	38.3	1 (reference)	1 (reference)
Yes	13	142	91.5	2.31 (1.33–4.01)^**^	2.47 (1.41–4.32)^**^
Diffuse connective disease					
No	424	10,884	38.9	1 (reference)	1 (reference)
Yes	8	197	40.6	1.00 (0.50–2.02)	–
Pneumonia					
No	304	8,410	36.1	1 (reference)	1 (reference)
Yes	128	2,671	47.9	1.25 (1.02–1.54)^*^	0.95 (0.76–1.18)
COPD					
No	165	5,673	29.0	1 (reference)	1 (reference)
Yes	267	5,408	49.3	1.62 (1.33–1.97)^***^	1.18 (0.96–1.45)
Diabetes					
No	360	10,149	35.4	1 (reference)	1 (reference)
Yes	72	932	77.2	2.05 (1.59–2.65)^***^	1.35 (1.04–1.76)^*^
Aspergillosis					
No	431	11,073	38.9	1 (reference)	1 (reference)
Yes	1	8	125	2.93 (0.41–20.86)	–
Candiasis					
No	431	11,077	38.9	1 (reference)	1 (reference)
Yes	1	4	250	6.51 (0.91–46.59)	–
Endemic mycoses					
No	432	11,081	38.9	1 (reference)	1 (reference)
Yes	0	0	0	–	–
Mounier-Kuhn					
No	432	11,081	38.9	1 (reference)	1 (reference)
Yes	0	0	0	–	–
Cystic fibrosis					
No	432	11,081	38.9	1 (reference)	1 (reference)
Yes	0	0	0	–	–
Hypertension					
No	216	8,031	26.8	1 (reference)	1 (reference)
Yes	216	3,050	70.8	2.52 (2.08–3.04)^***^	1.67 (1.35–2.07)^***^
Hyperlipidemia					
No	339	9,433	35.9	1 (reference)	1 (reference)
Yes	93	1,648	56.4	1.50 (1.19–1.89)^***^	1.13 (0.89–1.44)
Pulmonary embolism					
No	432	11,063	39.0	1 (reference)	1 (reference)
Yes	0	18	0	–	–
Depression					
No	428	11,014	38.8	1 (reference)	1 (reference)
Yes	4	67	59.7	1.44 (0.53–3.85)	–
Smoking					
Tobacco dependence					
No	432	11,058	39.0	1 (reference)	1 (reference)
Yes	0	23	0	–	–
Tobacco use disorder complicating pregnancy					
No	432	11,081	38.9	1 (reference)	1 (reference)
Yes	0	0	0	–	–
LABA					
Non-use	382	9,500	40.2	1 (reference)	1 (reference)
Use	50	1,581	31.6	0.76 (0.56–1.02)	–
LAMA					
Non-use	427	10,907	39.1	1 (reference)	1 (reference)
Use	5	174	28.7	0.72 (0.29–1.74)	–
SABA					
Non-use	297	6,846	43.3	1 (reference)	1 (reference)
Use	135	4,235	31.8	0.72 (0.59–0.89)^**^	0.67 (0.54–0.83)^***^
SAMA					
Non-use	336	8,257	40.6	1 (reference)	1 (reference)
Use	96	2,824	33.9	0.82 (0.65–1.03)	–
ICSs					
Non-use	341	8,365	40.7	1 (reference)	1 (reference)
Use	91	2,716	33.5	0.81 (0.64–1.02)	–
OSs					
Non-use	67	471	142.2	1 (reference)	1 (reference)
Use	365	10,610	34.4	0.24 (0.19–0.32)^***^	0.31 (0.23–0.41)^***^
Anti-arrhythmic					
Non-use	408	10,307	39.5	1 (reference)	1(reference)
Use	24	774	31.0	0.77 (0.51–1.17)	–
Alprazolam					
Non-use	328	7,816	41.9	1 (reference)	1 (reference)
Use	104	3,265	31.8	0.76 (0.61–0.95)^*^	0.87 (0.69–1.09)
Fluoxetine					
Non-use	432	11,081	38.9	1 (reference)	1 (reference)
Use	0	0	0	–	–
Fludiazepam					
Non-use	376	9,404	39.9	1 (reference)	1 (reference)
Use	56	1,677	33.3	0.85 (0.64–1.13)	–
Statins					
Non-use	397	9,593	41.4	1 (reference)	1 (reference)
Use	35	1,488	23.5	0.58 (0.41, 0.82)^**^	0.50 (0.35, 0.71)^**^
Anti-hypertensive drugs					
Diuretics					
Non-use	364	8,974	40.6	1 (reference)	1 (reference)
Use	68	2,106	32.3	0.78 (0.60, 1.01)	–
Beta blockers					
Non-use	370	8,847	41.8	1 (reference)	1 (reference)
Use	62	2,234	27.8	0.69 (0.53, 0.90)^**^	0.80 (0.60, 1.05)
Calcium channel blocker					
Non-use	328	7,837	41.9	1 (reference)	1 (reference)
Use	104	3,244	32.1	0.78 (0.63, 0.97)^*^	0.67 (0.53, 0.85)^**^
Angiotensin converting enzyme inhibitors					
Non-use	388	9,818	39.5	1 (reference)	1 (reference)
Use	44	1,263	34.9	0.89 (0.65–1.22)	–
Angiotensin II blockers					
Non-use	393	10,020	39.2	1 (reference)	1 (reference)
Use	39	1,061	36.8	0.94 (0.68, 1.31)	–

*BCAS cohort, Bronchiectasis-Asthma combination cohort; COPD, Chronic obstructive pulmonary disease*.

*PY, person-years; IR, incidence rate, per 1,000 person-years; HR, hazard ratio; CI, confidence interval; HR adjusted for BCAS cohort, gender, age, Rheumatoid arthritis, Pneumonia, COPD, Diabetes, Hypertension, Hyperlipidemia, SABAs, OSs, Alprazolam, Statins, Beta blocking blockers, and Calcium channel blockers*.

*LABAs/LAMAs, long-acting β2-agonist or muscarinic antagonist; SABAs/SAMAs, short-acting β2-agonist or muscarinic antagonist, steroids; ICSs, inhaled corticosteroid steroids; OSs, oral steroids; Beta blockers, cardioselective beta blockers (atenol, bisoprolol, metoprolol)*.

*–, Unable to calculate because of there are few or no events in with and without BCAS cohort*.

* *p < 0.05*,

** *p < 0.01*,

*** *p < 0.001*.

### Risk of HDS Among the BCAS Cohort and the Non-BCAS Cohort on Comorbidities and Medication

As shown in Table 3, 182 patients with HDS in the BCAS cohort and 250 patients with HDS in the non-BCAS cohort were included in this analysis. After adjustment for age, comorbidities, and medications, the BCAS cohort had a higher risk of HDS than the non-BCAS cohort among female (aHR = 1.42; 95% CI = 1.07–1.88), male (aHR = 2.39; 95% CI = 1.82–3.14), patients aged 20–39 years (aHR = 4.26; 95% CI = 1.38–13.2), patients aged 40–64 years (aHR = 1.57; 95% CI = 1.18–2.08), and patients over 65 years (aHR = 2.07; 95% CI = 1.55–2.76), patients with pneumonia (aHR = 2.39; 95% CI = 1.63–3.50), COPD (aHR = 2.15; 95% CI = 1.67–2.77), patients with diabetes (aHR = 1.84; 95% CI = 1.12–3.02), patients with hypertension (aHR = 1.87; 95% CI = 1.42–2.46), patients with hyperlipidaemia (aHR = 1.73; 95% CI = 1.12–2.67), patients using LABAs (aHR = 2.36; 95% CI = 1.25–4.43), patients using SABAs (aHR = 2.65; 95% CI = 1.87–3.75), patients using SAMAs (aHR = 2.66; 95% CI = 1.74–4.05), patients using ICSs (aHR = 2.53; 95% CI = 1.61–3.99), patients using OSs (aHR = 1.76; 95% CI = 1.43–2.18), patients using antiarrhythmic drugs (aHR = 9.88; 95% CI = 3.27–30.5), and patients using BZDs (alprazolam: aHR = 1.73; 95% CI = 1.15–2.58). All medications were associated with an increased risk of HDS, except fludiazepam (aHR = 1.33; 95% CI = 0.73–2.40).

**Table 3:** Incidence rate and hazard ratio of ischemic stroke or heart-disease between two cohorts stratified by gender, age, comorbidities and drug use after propensity matching.

	**BCAS cohort**							
	**No**			**Yes**				
	**Event**	**PY**	**IR**	**Event**	**PY**	**IR**	**Crude HR (95% CI)**	**Adjusted HR (95% CI)**
Gender								
Female	130	4,018	32.35	83	2,041	40.66	1.23 (0.94–1.63)	1.42 (1.07–1.88)^*^
Male	120	3,531	33.9	99	1,491	66.39	1.97 (1.51–2.57)^***^	2.39 (1.82–3.14)^***^
Age								
<20	2	577	3.46	0	284	0	–	–
20–39	7	1,429	4.89	9	661	13.61	2.65 (0.98–7.13)	4.26 (1.38–13.2)^*^
40–64	109	3,380	32.24	94	1,890	49.73	1.53 (1.16–2.02)^**^	1.57 (1.18–2.08)^***^
≥65	132	2,163	61.02	79	697	113.34	1.85 (1.40–2.45)^***^	2.07 (1.55–2.76)^***^
Comorbidity								
Pulmonary tuberculosis								
No	224	6,947	32.24	164	3,185	51.49	1.58 (1.29–1.94)^***^	1.91 (1.56–2.34)^***^
Yes	26	602	43.18	18	247	72.87	1.24 (0.67–2.26)	1.57 (0.82–2.99)
Non-tuberculosis mycobacterium								
No	249	7,515	33.13	181	3,522	51.39	1.54 (1.27–1.87)^***^	1.84 (1.52–2.24)^***^
Yes	1	34	29.41	1	10	100	1.73 (0.10–27.89)	–
Rheumatoid arthritis								
No	241	7,460	32.30	178	3,479	51.16	1.57 (1.29–1.91)^***^	1.87 (1.54–2.28)^***^
Yes	9	89	101.12	4	53	75.47	0.78 (0.22–2.70)	–
Diffuse connective disease								
No	245	7,396	33.12	179	3,488	51.31	1.54 (1.27–1.87)^***^	1.86 (1.52–2.26)^***^
Yes	5	153	32.67	3	44	68.18	2.17 (0.51–9.20)	0.83 (0.06–10.37)
Pneumonia								
No	180	5,766	31.21	124	2,644	46.89	1.49 (1.18–1.87)^***^	1.63 (1.29–2.05)^***^
Yes	70	1,783	39.25	58	888	65.31	1.68 (1.19–2.38)^**^	2.39 (1.63–3.50)^***^
COPD								
No	98	3,744	26.17	67	1,929	34.73	1.31 (0.96–1.79)	1.37 (0.99–1.89)
Yes	152	3,805	39.94	115	1,603	71.74	1.78 (1.40–2.28)^***^	2.15 (1.67–2.77)^***^
Diabetes								
No	210	6,932	30.29	150	3,217	46.62	1.53 (1.24–1.88)^***^	1.81 (1.46–2.25)^***^
Yes	40	617	64.82	32	315	101.58	1.56 (0.98–2.50)	1.84 (1.12–3.02)^**^
Aspergillosis								
No	250	7,549	33.11	181	3,524	51.36	1.54 (1.27–1.87)^***^	1.87 (1.54–2.27)^***^
Yes	0	0		1	8	125	–	–
Candiasis								
No	249	7,547	32.99	182	3,531	51.54	1.55 (1.28–1.88)^***^	1.88 (1.55–2.28)^***^
Yes	1	2	500	0	1	0	–	–
Endemic mycoses								
No	250	7,549	33.11	182	3,532	51.52	1.54 (1.28–1.87)^***^	1.87 (1.54–2.27)^***^
Yes	0	0	0	0	0	0	–	–
Mounier-Kuhn								
No	250	7,549	33.11	182	3,532	51.52	1.54 (1.28–1.87)^***^	1.87 (1.54–2.27)^***^
Yes	0	0	0	0	0	0	–	–
Cystic fibrosis								
No	250	7,549	33.11	182	3,532	51.52	1.54 (1.28–1.87)^***^	1.87 (1.54–2.27)^***^
Yes	0	0	0	0	0	0	–	–
Hypertension								
No	124	5,385	23.02	92	2,645	34.78	1.49 (1.14–1.96)^**^	1.81 (1.37–2.40)^***^
Yes	126	2,164	58.22	90	887	101.46	1.73 (1.32–2.27)^***^	1.87 (1.42–2.46)^***^
Hyperlipidemia								
No	192	6,355	30.21	147	3,078	47.75	1.57 (1.26–1.94)^***^	1.82 (1.46–2.27)^***^
Yes	58	1,194	48.57	35	454	77.09	1.56 (1.02–2.37)^*^	1.73 (1.12–2.67)^*^
Pulmonary embolism								
No	250	7,530	33.20	182	3,532	51.52	1.54 (1.27–1.87)^***^	1.85 (1.52–2.24)^***^
Yes	0	19	0	0	0	0	–	–
Depression								
No	248	7,498	33.07	180	3,516	51.19	1.54 (1.27–1.86)^***^	1.85 (1.52–2.24)^***^
Yes	2	51	39.21	2	16	125	2.02 (0.28–14.41)	–
Smoking								
Tobacco dependence								
No	250	7,534	33.18	182	3,524	51.64	1.55 (1.28–1.87)^***^	1.85 (1.52–2.25)^***^
Yes	0	15	0	0	8	0	–	–
Tobacco use disorder complicating pregnancy								
No	250	7,549	33.11	182	3,532	51.52	1.54 (1.28–1.87)^***^	1.85 (1.52–2.25)^***^
Yes	0	0	0	0	0	0	–	–
Drug use								
LABA								
Non-use	228	6,658	34.24	154	2,842	54.18	1.57 (1.28–1.93)^***^	1.83 (1.49–2.25)^***^
Use	22	891	24.69	28	690	40.57	1.65 (0.94–2.88)	2.36 (1.25–4.43)^*^
LAMA								
Non-use	249	7,452	33.41	178	3,455	51.51	1.53 (1.26–1.86)^***^	1.83 (1.50–2.22)^***^
Use	1	97	10.30	4	77	51.94	4.92 (0.54–44.34)	–
SABA								
Non-use	186	4,737	39.26	111	2,109	52.63	1.32 (1.05–1.68)^*^	1.62 (1.27–2.05)^***^
Use	64	2,812	22.75	71	1,423	49.89	2.18 (1.55–3.06)^***^	2.65 (1.87–3.75)^***^
SAMA								
Non-use	207	5,677	36.46	129	2,579	50.01	1.36 (1.09–1.69)^**^	1.69 (1.35–2.12)^***^
Use	43	1,872	22.97	53	953	55.61	2.40 (1.61–3.60)^***^	2.66 (1.74–4.05)^***^
ICSs								
Non-use	211	5,976	35.30	130	2,389	54.41	1.53 (1.23–1.90)^***^	1.72 (1.38–2.14)^***^
Use	39	1,573	24.79	52	1,143	45.49	1.83 (1.21–2.78)^**^	2.53 (1.61–3.99)^***^
OSs								
Non-use	38	352	107.95	29	119	243.69	2.05 (1.26–3.34)^**^	2.40 (1.44–3.99)^**^
Use	212	7,197	29.45	153	3,413	44.82	1.52 (1.23–1.87)^***^	1.76 (1.43–2.18)^***^
Anti-arrhythmic								
Non-use	240	7,026	34.15	168	3,281	51.20	1.49 (1.22–1.82)^***^	1.72 (1.41–2.11)^***^
Use	10	523	19.12	14	251	55.77	3.01 (1.32–6.81)^**^	9.88 (3.27–30.5)^***^
Alprazolam								
Non-use	189	5,349	35.33	139	2,467	56.34	1.58 (1.27–1.97)^***^	1.88 (1.50–2.34)^***^
Use	61	2,200	27.72	43	1,065	40.37	1.44 (0.98–2.14)	1.73 (1.15–2.58)^**^
Fluoxetine								
Non-use	250	7,549	33.11	182	3,532	51.52	1.54 (1.28–1.87)^***^	1.86 (1.53–2.26)^***^
Use	0	0	0	0	0	0	–	–
Fludiazepam								
Non-use	215	6,398	33.60	161	3,006	53.55	1.59 (1.29–1.95)^***^	1.94 (1.57–2.39)^***^
Use	35	1,151	30.40	21	526	39.92	1.30 (0.75–2.23)	1.33 (0.73–2.40)

*BCAS cohort, Bronchiectasis-Asthma combination cohort; COPD, Chronic obstructive pulmonary disease; PY, person-years; IR, incidence rate, per 1,000 person-years; HR, hazard ratio; CI, confidence interval*.

*HR adjusted for BCAS cohort, gender, age, Rheumatoid arthritis, Pneumonia, COPD, Diabetes, Hypertension, Hyperlipidemia, SABAs, OSs, Alprazolam, Statins, Beta blockers, and Calcium channel blockers*.

*LABAs/LAMAs, long-acting β2-agonist or muscarinic antagonist; SABAs/SAMAs, short-acting β2-agonist or muscarinic antagonist, steroids; ICSs, inhaled corticosteroid steroids; OSs, oral steroids; Beta blockers, cardioselective beta blockers (atenol, bisoprolol, metoprolol)*.

*–, Unable to calculate because of there are few or no events in with and without BCAS cohort*.

* *p < 0.05*,

** *p < 0.01*,

*** *p < 0.001*.

### Comparison Between Different Durations From the Last Day of Medication Use to HDS Occurrence Among the BCAS Cohort and the Non-BCAS Cohort

Table 4 shows that relative to the non-BCAS cohort, the BCAS cohort had a significantly higher risk of HDS between the final day of use and the HDS event. The aHRs and 95 % CI of the patients in the Table 4 display below: patients with LABAs > 90 days (aHRs = 4.58; 95% CI = 1.71–12.3), SABAs ≦30 days (aHRs = 2.80; 95% CI = 1.81–4.33), SAMA ≦30 days (aHRs = 3.00; 95% CI = 1.78–5.04), ICSs > 90 days (aHRs = 4.61; 95% CI = 2.18–9.76), OSs ≦30 days (aHRs = 1.80; 95% CI = 1.43–2.25), antiarrhythmic drugs ≦30 days (aHRs = 6.69; 95% CI = 1.55–28.8), and alprazolam ≦30 days (aHRs = 1.78; 95% CI = 1.09–2.93); 30–90 days (aHRs = 777.8; 95% CI = 1.34–451590.0).

**Table 4:** Incidence rate and hazard ratio of ischemic stroke or heart-disease between two cohorts stratified by current, recent and past use.

	**BCAS cohort**							
	**No**			**Yes**				
	**Event**	**PY**	**IR**	**Event**	**PY**	**IR**	**Crude HR (95%CI)**	**Adjusted HR (95%CI)**
Drug-use days		7,549			3,532			
LABA								
Non-use	228	6,658	34.24	154	2,842	54.18	1.57 (1.28–1.93)^***^	1.86 (1.51, 2.29)^***^
Current use (≤ 30 d)	14	319	43.88	12	173	69.36	1.64 (0.76–3.56)	1.10 (0.38, 3.15)
Recent use (30–90 d)	1	41	24.39	1	27	37.03	1.50 (0.09–23.98)	–
Past use (>90 d)	7	531	13.18	15	490	30.61	2.29 (0.93–5.63)	4.58 (1.71, 12.3)^**^
LAMA								
Non-use	249	7,452	33.41	178	3,455	51.51	1.53 (1.26–1.86)^***^	1.85 (1.52, 2.25)^***^
Current use (≤ 30 d)	0	51	0	3	44	68.18	–	–
Recent use (30–90 d)	0	3	0	0	0		–	–
Past use (>90 d)	1	43	23.25	1	33	30.30	1.52 (0.09–24.57)	–
SABA								
Non-use	186	4,737	39.26	111	2,109	52.63	1.32 (1.05–1.68)^*^	1.62 (1.27, 2.05)^***^
Current use (≤ 30 d)	40	778	51.41	47	350	134.28	2.58 (1.69–3.93)^***^	2.80 (1.81, 4.33)^***^
Recent use (30–90 d)	1	99	10.10	2	42	47.61	3.65 (0.32–40.75)	1.58 (0.33, 7.59)
Past use (>90 d)	23	1,935	11.88	22	1,031	21.33	1.80 (1.00–3.23)^*^	1.73 (0.79, 3.81)
SAMA								
Non-use	207	5,677	36.46	129	2,579	50.01	1.36 (1.09–1.69)^**^	1.70 (1.36, 2.13)^***^
Current use (≤ 30 d)	27	674	40.05	37	262	141.22	3.49 (2.12–5.74)^***^	3.00 (1.78, 5.04)^***^
Recent use (30–90 d)	1	78	12.82	0	25	0	–	–
Past use (>90 d)	15	1,120	13.39	16	666	24.02	1.79 (0.88–3.62)	0.48 (0.14, 1.65)
ICSs								
Non-use	211	5,976	35.30	130	2,389	54.41	1.53 (1.23–1.90)^***^	1.75 (1.40, 2.18)^***^
Current use (≤ 30 d)	20	415	48.19	23	208	110.57	2.35 (1.29–4.30)^**^	1.45 (0.76, 2.77)
Recent use (30–90 d)	1	58	17.24	2	43	46.51	2.87 (0.26–31.75)	–
Past use (>90 d)	18	1,100	16.36	27	892	30.26	1.84 (1.01–3.35)^*^	4.61 (2.18, 9.76)^***^
OSs								
Non-use	38	352	107.95	29	118	245.76	2.05 (1.26–3.34)^**^	2.40 (1.44–3.99)^***^
Current use (≤ 30 d)	175	2,272	77.02	141	999	141.14	1.83 (1.46–2.28)^***^	1.80 (1.43–2.25)^***^
Recent use (30–90 d)	6	680	8.82	0	352	0	–	–
Past use (>90 d)	31	4,245	7.30	12	2,063	5.81	0.78 (0.40–1.53)	1.51 (0.76–2.99)
Anti-arrhythmic								
Non-use	240	7,026	34.15	168	3,281	51.20	1.49 (1.22–1.82)^***^	1.80 (1.47–2.20)^***^
Current use (≤ 30 d)	6	172	34.88	5	46	108.69	4.24 (1.12–16.0)^*^	6.69 (1.55, 28.8)^*^
Recent use (30–90 d)	2	54	37.03	2	14	142.85	3.08 (0.43–22.01)	–
Past use (>90 d)	2	297	6.73	7	191	36.64	5.50 (1.13–26.69)^*^	–
Alprazolam								
Non-use	189	5,349	35.33	139	2,467	56.34	1.58 (1.27–1.97)^***^	1.88 (1.50–2.34)^***^
Current use (≤ 30 d)	35	385	90.90	23	180	127.77	1.41 (0.83–2.40)	1.78 (1.09–2.93)^*^
Recent use (30–90 d)	2	128	15.62	4	56	71.42	4.00 (0.72–22.09)	777.8 (1.34–451590.0)^*^
Past use (>90 d)	24	1,687	14.22	16	829	19.30	1.33 (0.70–2.51)	1.57 (0.55–4.46)
Fluoxetine								
Non-use	250	7,549	33.11	182	3,532	51.52	1.54 (1.28–1.87)^***^	1.86 (1.53–2.26)^***^
Current use (≤ 30 d)	0	0	0	0	0	0	–	–
Recent use (30–90 d)	0	0	0	0	0	0	–	–
Past use (>90 d)	0	0	0	0	0	0	–	–
Fludiazepam								
Non-use	215	6,398	33.60	161	3,006	53.55	1.59 (1.29–1.95)^***^	1.94 (1.57–2.39)^***^
Current use (≤ 30 d)	11	129	85.27	10	53	188.67	2.14 (0.90–5.08)	1.39 (0.75–2.59)
Recent use (30–90 d)	1	28	35.71	0	46	0	–	–
Past use (>90 d)	23	994	23.13	11	427	25.76	1.10 (0.54–2.27)	1.29 (0.42–4.01)

*BCAS cohort, Bronchiectasis-Asthma combination cohort; COPD, Chronic obstructive pulmonary disease*.

*PY, person-years; IR, incidence rate, per 1,000 person-years; HR, hazard ratio; CI, confidence interval*.

*HR adjusted for BCAS cohort, gender, age, Rheumatoid arthritis, Pneumonia, COPD, Diabetes, Hypertension, Hyperlipidemia, SABAs, OSs, Alprazolam, Statins, Beta blockers and Calcium channel blockers*.

*LABAs/LAMAs, long-acting β2-agonist or muscarinic antagonist; SABAs/SAMAs, short-acting β2-agonist or muscarinic antagonist, steroids; ICSs, inhaled corticosteroid steroids; OSs, oral steroids; Beta blockers, cardioselective beta blockers (atenol, bisoprolol, metoprolol)*.

*–, Unable to calculate because of there are few or no events in with and without BCAS cohort*.

* *p < 0.05*,

** *p < 0.01*,

*** *p < 0.001*.

However, for LABAs (≦30 days), SABA (30–90days, >90days), SAMAs (>90 days), ICSs (≦30 days), OSs (>90 days), alprazolam (>90 days), fludiazepam (≦30 days, >90 days) were not associated with the HDS.

### Comparison of HDS for Different Cumulative Daily Defined Doses of Medication in the BCAS Cohort and Non-BCAS Cohort

As shown in Table 5, relative to the non-BCAS cohort, a significant higher risk of HDS was observed for the cumulative daily defined dose (cDDD) of 416–2,300 DDDs for LABAs (aHR = 18.7; 95% CI = 1.29–272.7); >165 DDDs for SABAs [aHR = 3.31, 95% (1.65–6.65)]; ≤ 415, 415–1500, >1500 DDDs for ICSs (aHR = 5.02; 95% CI = 1.76–14.3; aHR = 2.58; 95% CI = 1.22–5.46; and aHR = 3.34; 95% CI = 1.40–7.97, respectively); ≦15, 16–155, and >155 DDDs for OSs (aHR = 2.28; 95% CI = 1.43–3.62; aHR = 1.90; 95% CI = 1.28–2.81; and aHR = 1.95; 95% CI = 1.26–3.02, respectively); and 6–30 DDDs for alprazolam (aHR = 2.31; 95% CI = 1.09–4.89).

**Table 5:** Incidence rate and hazard ratio of ischemic stroke or heart-disease between two cohorts stratified by cumulative dose of drug.

	**BCAS cohort**							
	**No**			**Yes**				
	**Event**	**PY**	**IR**	**Event**	**PY**	**IR**	**Crude HR (95%CI)**	**Adjusted HR (95%CI)**
Cumulative dose of drug								
LABA (DDD)								
Non-use	228	6,829	33.38	132	2,791	47.29	1.41 (1.14–1.75)^**^	1.76 (1.43–2.16)^***^
≤ 415	5	124	40.32	9	116	77.58	1.83 (0.61–5.49)	2.95 (0.22–38.8)
416–2,300	11	407	27.02	23	315	73.01	2.71 (1.32–5.57)^**^	18.7 (1.29,.272.7)^*^
>2,300	6	189	31.74	18	310	58.06	1.84 (0.73–4.64)	11.4 (0.45–10.5)
LAMA(DDD)								
Non-use	238	7,297	32.61	156	3,292	47.38	1.44 (1.18–1.77)^***^	1.70 (1.37–2.12)^***^
≤ 30	4	69	57.97	10	97	103.09	1.71 (0.53–5.46)	3.78 (0.37, 38.5)
31–210	4	96	41.66	8	61	131.14	2.66 (0.80–8.88)	2.97 (1.36, 6.51)
>210	4	87	45.97	8	82	97.56	2.00 (0.60–6.69)	3.11 (0.90, 10.8)
SABA (DDD)								
Non-use	199	6,111	32.56	98	2,388	41.03	1.26 (0.99–1.60)	1.57 (1.23–2.01)^***^
≤ 1	23	631	36.45	19	274	69.34	1.88 (1.02–3.46)^*^	1.29 (0.62–2.69)
2–165	14	350	40	31	371	83.55	2.03 (1.08–3.82)^*^	1.79 (0.91–3.55)
>165	14	457	30.63	34	499	68.13	2.22 (1.19–4.14)^*^	3.31 (1.65–6.65)^***^
SAMA (DDD)								
Non-use	224	6,934	32.30	128	2,959	43.25	1.33 (1.07–1.66)^**^	1.64 (1.32–2.05)^***^
≤ 1.5	0	0		0	0		–	2.18 (1.17–4.09)^***^
1.6–5	22	453	48.56	30	342	87.71	1.79 (1.03–3.11)^*^	–
>5	4	162	24.69	24	231	103.89	3.83 (1.32–11.06)^*^	7.91 (1.76–35.6)^***^
ICSs (DDD)								
Non–use	215	6,235	34.48	108	2,354	45.87	1.33 (1.05–1.68)^*^	1.54 (1.22–1.95)^***^
≤ 415	10	461	21.69	11	199	55.27	2.55 (1.08–6.02)^*^	5.02 (1.76–14.3)^**^
416–1,500	13	508	25.59	32	511	62.62	2.36 (1.23–4.50)^**^	2.58 (1.22–5.46)^*^
>1,500	12	345	34.78	31	468	66.23	1.81 (0.93–3.54)	3.34 (1.40–7.97)^**^
OSs (DDD)								
Non-use	102	1,738	58.68	46	452	101.76	1.72 (1.21–2.44)^**^	2.77 (1.44–2.97)^***^
≤ 15	55	2,136	25.74	32	683	46.85	1.81 (1.17–2.80)^**^	2.28 (1.43–3.62)^**^
16–155	55	2,012	27.33	51	962	53.01	1.90 (1.30–2.79)^***^	1.90 (1.28–2.81)^**^
>155	38	1,663	22.85	53	1,435	36.93	1.62 (1.07–2.47)^*^	1.95 (1.26–3.02)^**^
Anti-arrhythmia								
Non-use	244	7,398	32.98	171	3,416	50.05	1.51 (1.24–1.84)^***^	1.81 (1.49, 2.21)^***^
≤ 35	1	64	15.62	8	88	90.90	5.80 (0.72–46.73)	–
36–65	0	0		0	0		–	–
>65	5	87	57.47	3	28	107.14	1.41 (0.33–6.07)	–
Alprazolam (DDD)								
Non-use	189	5,349	35.33	139	2,469	56.29	1.58 (1.27–1.97)^***^	1.88 (1.50–2.34)^***^
≤ 5	22	687	32.02	7	229	30.56	0.96 (0.41–2.25)	1.70 (0.64–4.48)
6–30	19	742	25.60	19	393	48.34	1.92 (1.01–3.62)^*^	2.31 (1.09–4.89)^*^
>30	20	771	25.94	17	441		1.56 (0.81–2.99)	1.60 (0.78–3.29)
Fluoxetine								
Non-use	250	7,549	33.11	182	3,532	51.52	1.54 (1.28–1.87)^***^	1.86 (1.53–2.26)^***^
≤	0	0	0	0	0	0	–	–
-	0	0	0	0	0	0	–	–
>	0	0	0	0	0	0	–	–
Fludiazepam								
Non-use	215	6,398	33.60	161	3,006	53.55	1.59 (1.29–1.95)^***^	1.94 (1.57–2.39)^***^
≤ 5	14	401	34.91	4	142	28.16	0.78 (0.25–2.40)	1.27 (0.33–4.82)
6–20	9	351	25.64	8	191	41.88	1.61 (0.62–4.18)	1.22 (0.35–4.17)
>20	12	399	30.07	9	193	46.63	1.56 (0.66–3.72)	2.43 (0.90–6.55)

*BCAS cohort, Bronchiectasis-Asthma combination cohort; COPD, Chronic obstructive pulmonary disease; PY, person-years; IR, incidence rate, per 1,000 person-years; HR, hazard ratio; CI, confidence interval*.

*HR adjusted for BCAS cohort, gender, age, Rheumatoid arthritis, Pneumonia, COPD, Diabetes, Hypertension, Hyperlipidemia, SABAs, OSs, Alprazolam, Statins, Beta blockers, and Calcium channel blockers*.

*LABAs/LAMAs, long-acting β2-agonist or muscarinic antagonist; SABAs/SAMAs, short-acting β2-agonist or muscarinic antagonist, steroids; ICSs, inhaled corticosteroid steroids; OSs, oral steroids; Beta blockers, cardioselective beta blockers (atenol, bisoprolol, metoprolol)*.

*–, Unable to calculate because of there are few or no events in with and without BCAS cohort*.

* *p < 0.05*,

** *p < 0.01*,

*** *p < 0.001*.

However, there were not associated with the risk of the HDS for LABAs at ≤ 415 DDDs and >2,300 DDDs, LAMA at any dose, SABAs at ≤ 1 DDDs and 2–165 DDDs, alprazolam at ≤ 5 and >30 DDDs, and fludiazepam at ≤ 5, >6–20, and >20 DDDs.

**Figure 3:**
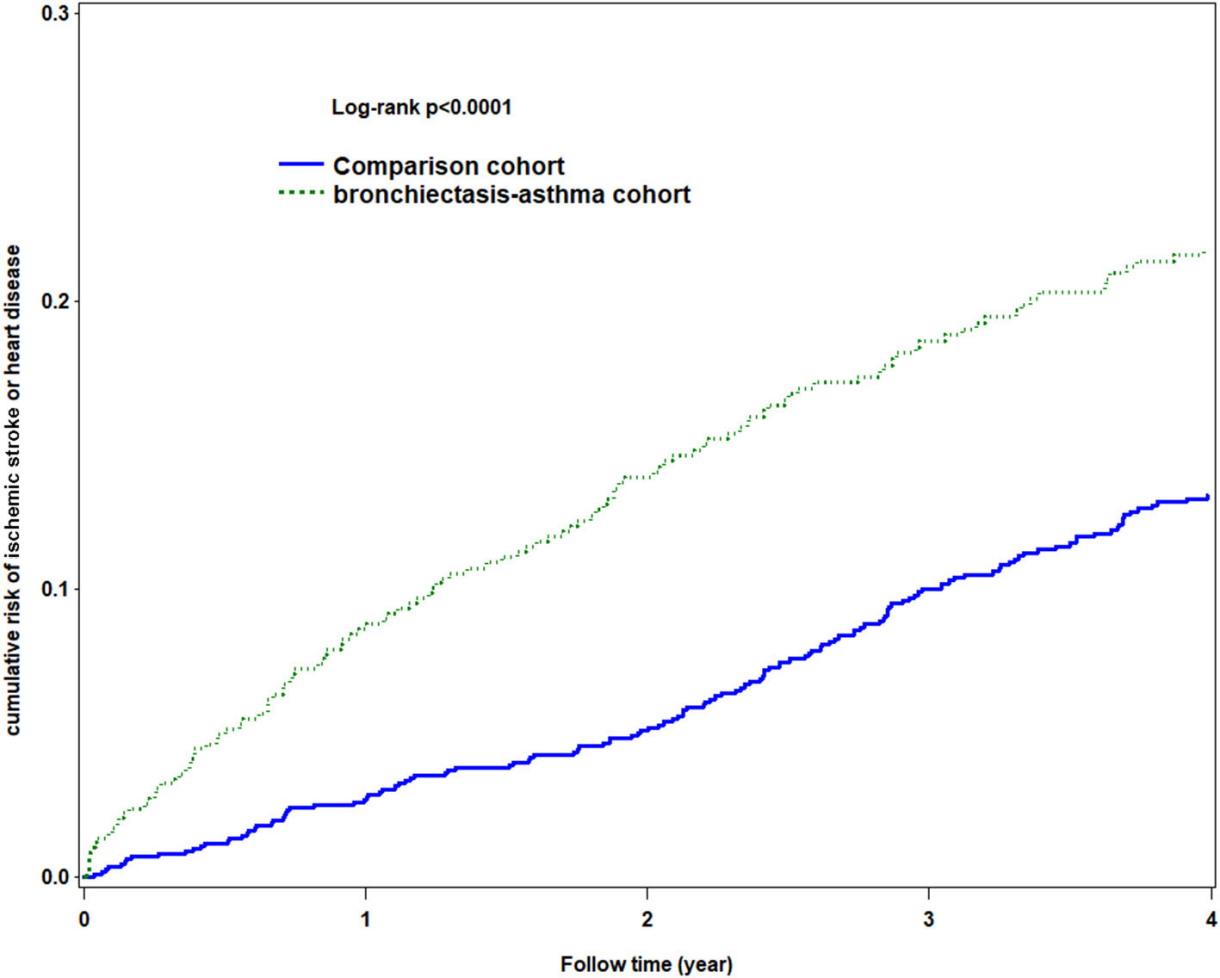
Using Kaplan-Meier survival statistics, it showed crude overall survival curves by with and without bronchiectasis-asthma combination cohort (log-rank *P* < 0.0001).

The Kaplan–Meier analysis for the cumulative incidence of HDS revealed significant differences between the BCAS cohort and the non-BCAS cohort (log-rank test, *p* < 0.0001) as being statistically significant in HDS (Figure 3).

### Validation of Bronchiectasis With Asthma

**Figure 4:**
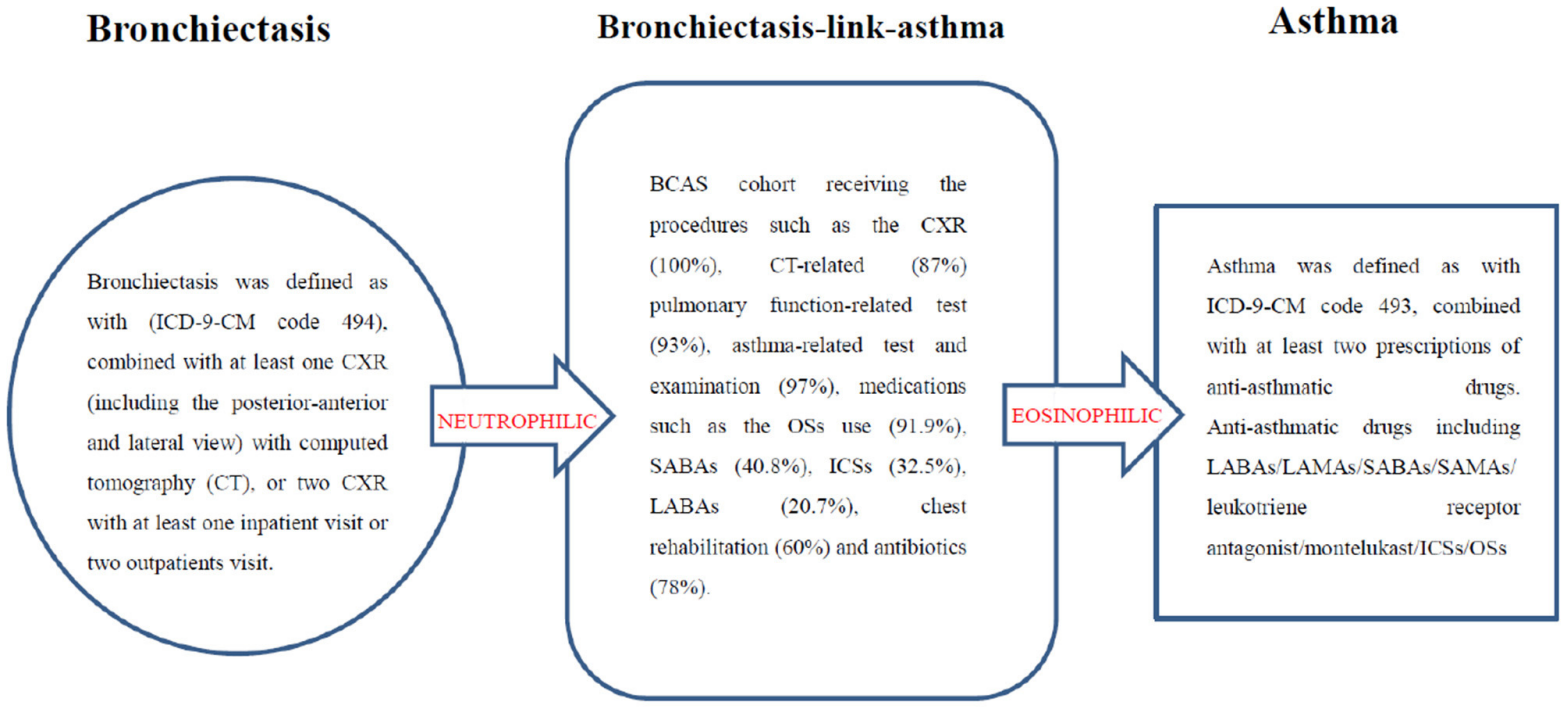
Validation of bronchiectasis-asthma combination.

Patients with BCAS cohort were derived from the bronchiectasis, asthma and COPD group presenting as the (6: bronchiectasis and asthma combination, BCAS) or (7: BCAS and COPD combination, BCAOS) in the general population (predominant BCAS (Figure 4).

## Summary Findings of Results

### Immortal Time Bias

To resolve the immortal time bias in this observational study, we established a 1-year confirmation period (14). Users were defined as patients who needed to start using medications and had at least one prescription and received treatment for at least 28 days within 1 year after BCAS cohort diagnosis. Non-users were defined as patients who did not receive a prescription for these drugs and were not treated for at least 28 days within 1 year after BCAS cohort diagnosis (Table 6).

**Table 6:** Summary findings of results.

	**A**	**B**	**Past**	**Recent**	**Current**	**High**	**Medium**	**Low**
LABAs	+		+		0	0	+	0
SABAs	+	–	0		+	+	0	0
LAMAs						0	0	0
SAMAs	+		0		+	+		+
ICSs	+		+		0	+	+	+
OSs	+	–	0		+	+	+	+
Anti-Arrhythmic	+				+			
Alprazolam	+	0	0	+	+	0	+	0
Fludiazepam	0		0		0	0	0	0
Statins		–						
Beta blockers: cardioselective		0						
Calcium channel blockers		–						

*A, In general, LABAs, SABAs, SAMAs, ICSs, OSs, antiarrhythmic drugs, and alprazolam were associated with a higher risk of HDS. LAMAs Fludiazepam were not associated with increased HDS risk*.

*B, Using patients who were not taking medication as the reference group, SABAs, OSs, Statins, and CCBs were associated with an attenuated risk of HDS*.

*+, increased risk; –, decreased risk; 0, no association with risk*.

*LABAs, long-acting β2 agonists; LAMAs, long-acting muscarinic antagonists; SABAs, short-acting β2 agonists; SAMAs, short-acting muscarinic antagonists; ICSs, inhaled corticosteroid steroids; OSs, oral steroids; CCBs, calcium channel blockers*.

Under a multiple disciplinary team, the pay-for-performance (P4P) of asthma including an initial visit for new patients, outpatient care and hospitalization, first prescription, emergency visits, drug refill prescriptions, and providers for producing an improvement in performance based on quality measures was determined (14, 15). This strict policy helped us to avoid immortal time bias in this study (16).

### Statins, Beta Blockers, Angiotensin-Converting Enzyme Inhibitors Angiotensin II-Receptor Blockers Use and Target Level for Hypertension, Diabetes, Low Density Lipoprotein-Cholesterol

Oxidative stress has been implicated in many pathophysiological conditions in the HDS, including hyperlipidemia, hypertension, and diabetes (17). These diseases associated with the higher risk of HDS in the BCAS cohort (Table 3) (18). The statins, beta blockers, renin-angiotensin system (RAS) inhibitors (e.g., ACEi, angiotensin II-receptor blockers, ARBs) with anti-inflammatory and oxidative stress effects (19). Experimental studies have shown reciprocal relationships between insulin resistance and endothelial dysfunction. Hyperlipidemia and hypertension have a synergistic deleterious effect on insulin resistance and endothelial dysfunction. Unregulated RAS is a key factor in the pathogenesis of atherosclerosis and hypertension. Various strategies with different classes of antihypertensive medications to reach target goals have failed to attenuate the residual HDS further. Of interest, treating hyperlipidemia with statins in hypertensive patients are associated with the lower HDS risk further (20). In previous study, statins therapy are associated with the higher risk for insulin resistance and type 2 diabetes mellitus. Fortunately, RAS inhibitors attenuate the endothelial dysfunction and risk of insulin resistance (21). In this regard, combined therapy with statins and RAS inhibitors not only demonstrates additive/synergistic effects on endothelial dysfunction and insulin resistance but also lowering cholesterol levels and blood pressure (BP) when compared with either monotherapy in patients having hypertension, hyperlipidemia (22).

Meanwhile, increased carotid intima-media thickness (CIMT) is associated with an increased risk for ischemic stroke (23). Calcium channel blockers (CCBs) and RAS inhibitors such as ARBs have a role for improving the nitric oxide production, modulating the oxidative stress, and attenuating the risk of CIMT in patients with hypertension (24). Thus, ARBs and CCBs use were associated with the lower risk of HDS such as ischemic stroke. Altogether, combined therapy with the statins and RAS inhibitors/CCBs may be the optimal management strategies in patients with hypertension, hyperlipidemia, diabetes to prevent HDS (25). In recent Taiwan NHIRD study reveal that the combined these cardioprotective drugs-statins, cardioselective beta-blockers, RAS inhibitors and CCBs have benefits for the HDS among the asthma or COPD support these speculations (26, 27). In our study, the (statins, CCBs) users have the lower risk of the HDS, with patients not using (statins, CCBs) as reference. These results were in line with previous meta-analysis study (24).

The hypertension Taiwan guideline 2010 recommended the lowering of target BP to <130/80 mmHg for HDS (2015, <140/90 mmHg for stroke; <130/80 mmHg for coronary artery disease or diabetes) (28). In general, Taiwanese physicians follow the current hypertension treatment guidelines relatively well, a high success rate of 63% in achieving the BP goal of <140/90 mmHg in outpatient clinics of hospital among general population (29). Guidelines of diabetes care for glycemic control have consistently targeted hemoglobin A1c (HbA1c) values <7%, pointing to the HDS benefits of maintaining HbA1c in this range while remaining mindful of the risks of hypoglycemia (30). The lipid guidelines for high risk patients recommended pragmatic goals for low density lipoprotein-cholesterol (LDL-C) of <70 mg/dL (<100 mg/dl, 2000–2009) for those at highest HDS (31, 32). A P4P programme is a management strategy that encourages healthcare providers to deliver high quality of care, and helps the BCAS cohort with these comorbidities to receive the management under these guidelines such as HbA1c <7.0%, BP <140/90 mmHg, and LDL-C <100 mg/dL (33–35).

### Health Behavior Nutraceuticals Food Habits in Relation to the HDS

Nutraceuticals, functional foods and supplements with a serum LDL-C lowering effect, the possible mechanism including: (1) absorption inhibitors: plant sterols and stanols, soluble fiber, oat fibers, psyllium, probiotics; (2) LDL synthesis inhibition: red yeast rice, bergamot, artichoke; (3) LDL excretion improving: soy proteins, berberine, and green tea extracts (36–38). Thus, they could represent useful compounds that are associated with lower risk of HDS by acting parallel to statins or as adjuvants in case of drugs failure or in situations where statins cannot be used (39). When statins are not available such as intolerance, side effects, or patient preference. The nutraceuticals (e.g., Bergamot-Derived Polyphenolic Fraction) and functional food-related diet (e.g., Mediterranean diet supplemented with extra-virgin olive oil or nuts) may help us for solve these problems (36, 40, 41). Among foods, beetroot juice has the most convincing evidence of lowering the BP. Among nutrients, magnesium, potassium and vitamin C supplements were associated with the lower BP. Notably, the use of nutraceuticals should never substitute the one of conventional drugs, when their prescription is indicated by the international guidelines. However, physical activity, healthy diet, and nutraceuticals may play an auxiliary role for prevention of HDS (36, 38, 40).

The diabetes P4P program for caring patients with diabetes alone and diabetes with comorbid hypertension and hyperlipidemia from a single payer in Taiwan could help the BCAS cohort to improve the health behavior and food habits including poor dietary practices, physical inactivity, and cigarette smoking (13, 33, 34). The lifestyle measures that are recommended to lower HDS including salt restriction, alcohol limitation, body reduction, cessation of smoking, diet adaptation, and exercise adoption. The strict policy of the health behavior, food habits, and higher adherence of medications such as statins and CCBs among the BCAS cohort (about 10.8% of diabetes) receiving the chronic care program may help patients to achieve the target BP, HBA1c, and LDL-C (42, 43). These complementary and integrative therapies have a critical role for attenuating the risk of HDS in BCAS cohort with comorbidities such as hyperlipidemia.

## Discussion

To the best of our knowledge, this study is the first to investigate the relationship between BZDs and the risk of HDS between the BCAS cohort and the non-BCAS cohort in the English literature to date. This general population study revealed four major findings. First, BZDs such as fludiazepam even current use were not associated with a higher risk of HDS in the BCAS cohort comparing with the non-BCAS cohort. However, the (current, recent) use and medium dosage of alprazolam were associated with a higher risk of HDS. Second, steroids (past ICSs, current OSs, any dose ICSs/OSs) were associated with a higher risk of HDS, even at a low dose, in the BCAS cohort than in the non-BCAS cohort. In addition, with patients not using OSs as the reference group, the results revealed that OSs use was associated with a lower risk of HDS. Third, the high dosage and current use of SABAs were associated with a higher risk of HDS. However, with patients without using SABAs as the reference group, SABAs were associated with a lower risk of HDS. Forth, the current use of LABAs/ICSs were not associated with HDS.

Anxiety may contribute to a cross-reaction with central processing at the cortical and brain stem level and the autonomic nerves, changing the electrophysiology of the myocardium and leading to cardiac arrhythmia. Relieving anxiety may attenuate the risk of HDS, including cardiac arrhythmia and heart failure, in the BCAS cohort. Similar to that, Balon et al. reported that BZDs may be associated with the lower risk of HDS, such as coronary artery disease and heart failure (44, 45). Meanwhile, Huang et al. reported that the lower dose of BZDs provided neuroprotection (45–47). Furthermore, Patorno et al. revealed little to no increase in all-cause mortality associated with BZDs initiation in the general population (48). These findings indicate that BZDs are not associated with significant risk of HDS support our results. However, the current study suggests that the (current, recent) use of alprazolam is associated with a higher risk of HDS; a possible explanation for this is the rebound response of insomnia with the (current, recent) use of intermediate-acting alprazolam (49). Rebound insomnia is associated with a higher risk of HDS. Fludiazepam is long acting and has a lower withdrawal response, which may prevent rebound insomnia and was not associated with the risk of HDS (50).

The BCAS cohort involves the impairment of the immune system, and steroids aggravate immune deficiency accompanied by infection, which may lead to a higher risk of HDS (51). In addition, the systemic effects of steroids can promote hyperglycaemia, hypertension, and hyperlipidaemia, contributing to HDS development. According to Yao et al., the highest rates of GI bleeding, sepsis, and heart failure occurred within the first month after the initiation of steroid therapy, which is in line with our results (52). However, the adverse reaction to OSs is attenuated after 30 days of use (52–54). This finding may explain why the past use of OSs was not associated with the higher risk of HDS. Notably, general steroid use (past ICSs, current OSs, any dose ICSs/OSs) were associated with a higher risk of HDS, even at a low dose (52, 54).

In the BCAS cohort, poor lung function and quality scores are linked to higher levels of cytokines, eosinophils, and neutrophils compassion of the non-BCAS cohort (2, 3). The anti-inflammatory effects (55, 56) of bronchodilators (LABAs/ LAMAs, SABAs/SAMAs), steroids, and antiarrhythmic drugs are limited; thus, the effect of these drugs for ameliorating the progression of persistent artery stiffness was suboptimal. Therefore, compared with the non-BCAS cohort, the BCAS cohort who used bronchodilators (current or high SABAs/SAMAs, steroids), and antiarrhythmic drugs (current use) had higher risks of HDS (5, 11, 57). However, with patients not using (SABAs, OSs) as the reference group, (SABAs, OSs) use were associated with a lower risk of HDS. As mention before, the complementary and integrative therapies under multidisciplinary team may play an auxiliary role for helping these patients to change their lifestyles, increase their adherence to medications (58). For example, the overuse of SABAs is relatively low in Taiwan compared with that in other countries (15.9%, similar to Germany but lower than that in other European countries), indicating that well-trained teams may encourage the BCAS cohort who use (SABAs, OSs) to attend regular follow-up appointments, promoting continued care for hypertension, and a higher quality of life and thus attenuating the risk of HDS (59). Notably, we found the (current LABAs/ICSs, any dose LAMAs) use were not associated with the HDS. The current use of LABAs/ICSs (e.g., formoterol/budesonide) seem to be superior to current use of SABAs/OSs in select scenario such as avoiding the HDS in BCAS cohort with diabetes/hypertension. The recent Chen et al. study concluded the risk of HDS was associated with COPD patients with preexisting cardiovascular disease and history of frequent exacerbations rather than associated with the use of LABAs/ICSs support these speculations (60–63). However, these findings warrant further research.

In summary, because of the increased risk of HDS, the bronchodilators, antiarrhythmic drugs, and steroids could be used after evaluation of the benefit in the BCAS cohort and low doses was suggested (64). Steroids could be used only in select cases, even at low doses. BZDs such as fludiazepam are relatively safe; however, the current or recent use of alprazolam are associated with a high risk of HDS (65).

### Strengths

The medical records in the NHIRD are highly accurate, making this database a strong resource for population-based cardiovascular and stroke research (66, 67). Bronchodilators, steroids (ICSs and OSs), statins and antihypertensive drug use in Taiwan follows international guidelines. Furthermore, the NHIRD-based identification of asthma, COPD, and bronchiectasis-related diseases, such as PTB and pneumonia, has been validated in several recent reports (60, 68, 69). Therefore, this well-established method prevented potential biases in this study.

### Limitations

The limitations of this study include bias and confounding variables. First, the results of observational studies are not as accurate as those of randomized control trials (RCT). Therefore, we performed a propensity score matching analysis to address this point (70). However, this retrospective study is usually lower evidence than the RCT trials because a retrospective study is subject to have many unknown confounding factors such as the other health problems. Meanwhile, old records were not designed to be used for future studies (67). Second, the NHIRD provides no detailed information on patients regarding factors such as their lifestyle, body mass index (or obesity), habits (such as smoking and alcoholic drinking), physical activity, socioeconomic status, or family history; all of which are possible confounding factors in this study. Third, the registries in the NHI claims are primarily used for administrative billing and are not verified for scientific purposes. Forth, lack of individual laboratory data such as BP, HBA1c, LDL-C, cytokine level, imaging finding in the NHIRD may be the other study limitation.

Fifth, in the sensitivity analysis, wwe found that the (current LABAs, any dose LAMAs) use were not associated with the HDS. In contrast, Wang et al. reported new initiation of (LABAs, LAMAs) in patients with COPD is associated with an ~1.5-fold increased cardiovascular disease, irrespective of prior cardiovascular disease status and history of exacerbations (53). In this study, we also found that (SABAs at DDD > 165, SAMAs at DDD > 5, past LABAs) use were associated with higher risk of HDS. Therefore, primary effect of the (bronchodilators) on the HDS among BCAS cohort could not explain these different findings. Perhaps, the primary effect of the BCAS cohort, or the joint effect of the BCAS cohort and individual comorbidity, or the combination effect of the medications with the BCAS cohort and their comorbidities contributing to HDS in this study. Thus, when we interpret these results, we should take the other confounding factors such as comorbid-related HDS into account. Altogether, the effect of the bronchodilators on the risk HDS warrant further research.

## Conclusion

The bronchodilators, steroids, and antiarrhythmic drugs were associated with higher risk of HDS, even low dose use of steroids. However, the current use of LABAs/ICSs use were not associated with HDS. The use of the BZDs is relatively safe, except for the current or recent use of alprazolam. Notably, taking confounders into account is crucial in observational studies.

## Data Availability Statement

The datasets presented in this article are not readily available because the dataset used in this study is held by the Taiwan Ministry of Health and Welfare (MOHW). The Ministry of Health and Welfare must approve our application to access this data. Any researcher interested in accessing this dataset can submit an application form to the Ministry of Health and Welfare requesting access. Please contact the staff of MOHW (email: stcarolwu@mohw.gov.tw) for further assistance. All relevant data are within the paper. Requests to access the datasets should be directed to email: stcarolwu@mohw.gov.tw.

## Ethics Statement

This study was approved by the Research Ethics Committee of China Medical University and Hospital in Taiwan (Institutional Review Board permit number: CMUH104-REC2-115-AR2). Written informed consent for participation was not required for this study in accordance with the national legislation and the institutional requirements.

## Author Contributions

J-JY and C-HK: conception and design. C-HK: administrative support. All authors: collection and assembly of data, data analysis and interpretation, manuscript writing, final approval of manuscript, contributed to the article, and approved the submitted version.

## Funding

This study was supported in part by Taiwan Ministry of Health and Welfare Clinical Trial Center (MOHW110-TDU-B-212-124004), China Medical University Hospital (DMR-109-231, DMR-110-089, DMR-111-090, DMR-111-091), and Ministry of Science and Technology (MOST 110-2321-B-039-003). The funders had no role in the study design, data collection and analysis, the decision to publish, or preparation of the manuscript.

## Conflict of Interest

The authors declare that the research was conducted in the absence of any commercial or financial relationships that could be construed as a potential conflict of interest.

## Publisher's Note

All claims expressed in this article are solely those of the authors and do not necessarily represent those of their affiliated organizations, or those of the publisher, the editors and the reviewers. Any product that may be evaluated in this article, or claim that may be made by its manufacturer, is not guaranteed or endorsed by the publisher.

## Abbreviations

BCAS: bronchiectasis–asthma combination
LABA: long-acting beta2 agonist
LAMA: long-acting muscarinic antagonist
SABA: short-acting beta2 agonist
SAMA: short-acting muscarinic antagonists
aHR: adjusted hazard ratio
CI: confidence interval
ICSs: inhaled corticosteroids
OSs: oral steroids
BZDs: benzodiazepines
NHIRD: National Health Insurance Research Database
LHID: Longitudinal Health Insurance Database
ICD-9-CM, International Classification of Diseases, Ninth revision: Clinical Modification.

## References

[1] Simpson JL Grissell TV Douwes J Scott RJ Boyle MJ Gibson PG. Innate immune activation in neutrophilic asthma and bronchiectasis. Thorax. (2007) 62:211–8. 10.1136/thx.2006.061358 16844729 PMC2117164

[2] Crimi C Campisi R Cacopardo G Doria G Intravaia R Morena P. Mepolizumab efficacy in patients with severe eosinophilic asthma and bronchiectasis. Euro Resp J. (2019) 54:PA2528. 10.1183/13993003.congress-2019.PA252830881051

[3] Carpagnano GE Scioscia G Lacedonia D Curradi G Foschino Barbaro MP. Severe uncontrolled asthma with bronchiectasis: a pilot study of an emerging phenotype that responds to mepolizumab. J Asthma Allergy. (2019) 12:83–90. 10.2147/JAA.S196200 30881051 PMC6407514

[4] Martínez-García MÁ Sánchez CP Moreno RMG. The double-edged sword of neutrophilic inflammation in bronchiectasis. Euro Resp J. (2015) 46:898–900. 10.1183/13993003.00961-2015 26424521

[5] Polverino E Dimakou K Hurst J Martinez-Garcia MA Miravitlles M Paggiaro P. The overlap between bronchiectasis and chronic airway diseases: state of the art and future directions. Eur Respir J. (2018) 52:1800328. 10.1183/13993003.00328-2018 30049739

[6] Cilli A Uzer F Boztepe Z. Clinical effects of asthma and bronchiectasis coexistence. Chest. (2018) 154:19A. 10.1016/j.chest.2018.08.016

[7] Bendien SA van Loon-Kooij S Kramer G Huijgen W Altenburg J Ten Brinke A. Bronchiectasis in severe asthma: does it make a difference? Respiration. (2020) 99:1136–44. 10.1159/000511459 33321494

[8] Merkler AE Parikh NS Mir S Gupta A Kamel H Lin E. Risk of ischemic stroke in patients with coronavirus disease 2019 (COVID-19) vs patients with influenza. JAMA Neurol. (2020) 77:1–7. 10.1001/jamaneurol.2020.2730 32614385 PMC7333175

[9] Gao YH Liu SX Cui JJ Wang LY Yin KQ Wang L. Subclinical atherosclerosis in adults with steady-state bronchiectasis: a case-control study. Respir Med. (2018) 134:110–6. 10.1016/j.rmed.2017.11.024 29413496

[10] Liu X Luo W-T Li Y Li C-N Hong Z-S Chen H-L. Psychological status and behavior changes of the public during the COVID-19 epidemic in China. Infect Dis Poverty. (2020) 9:58. 10.1186/s40249-020-00678-3 32471513 PMC7256340

[11] Navaratnam V Root AA Douglas I Smeeth L Hubbard RB Quint JK. Cardiovascular outcomes after a respiratory tract infection among adults with non-cystic fibrosis bronchiectasis: a general population-based study. Ann Am Thorac Soc. (2018) 15:315–21. 10.1513/AnnalsATS.201706-488OC 29266966 PMC5880522

[12] Garth J Barnes JW Krick S. Targeting cytokines as evolving treatment strategies in chronic inflammatory airway diseases. Int J Mol Sci. (2018) 19:3402. 10.3390/ijms19113402 30380761 PMC6275012

[13] Kao Y-H Wu S-C. STROBE-compliant article: is continuity of care associated with avoidable hospitalization among older asthmatic patients? Medicine. (2016) 95:e4948. 10.1097/MD.0000000000004948 27661052 PMC5044922

[14] Shariff SZ Cuerden MS Jain AK Garg AX. The secret of immortal time bias in epidemiologic studies. J Am Soc Nephrol. (2008) 19:841–3. 10.1681/ASN.2007121354 18322159

[15] Wang J-Y Liu L-F. Health care utilization and medical costs for childhood asthma in Taiwan: using Taiwan National Health Insurance Research Database. Asia Pac Allergy. (2012) 2:167–71. 10.5415/apallergy.2012.2.3.167 22872818 PMC3406295

[16] Kiri VA Pride NB Soriano JB Vestbo J. Inhaled corticosteroids in chronic obstructive pulmonary disease: results from two observational designs free of immortal time bias. Am J Respir Crit Care Med. (2005) 172:460–4. 10.1164/rccm.200502-210OC 15901610

[17] Scicchitano P Cortese F Gesualdo M De Palo M Massari F Giordano P. The role of endothelial dysfunction and oxidative stress in cerebrovascular diseases. Free Radic Res. (2019) 53:579–95. 10.1080/10715762.2019.1620939 31106620

[18] Ciccone MM Cortese F Gesualdo M Scicchitano P Ricci G Carbonara S. Correlation among atherosclerosis, cardiac and respiratory function in subjects with cystic fibrosis. Minerva Med. (2018) 109:250–4. 10.23736/S0026-4806.18.05469-1 29332379

[19] Huang L Chen Z Ni L Chen L Zhou C Gao C. Impact of angiotensin-converting enzyme inhibitors and angiotensin receptor blockers on the inflammatory response and viral clearance in COVID-19 patients. Front Cardiovasc Med. (2021) 8:710946. 10.3389/fcvm.2021.710946 34490373 PMC8416906

[20] Stone NJ Robinson JG Lichtenstein AH Merz CNB Blum CB Eckel RH. 2013 ACC/AHA guideline on the treatment of blood cholesterol to reduce atherosclerotic cardiovascular risk in adults. Circulation. (2014) 129:S1–S45. 10.1161/01.cir.0000437738.63853.7a 24222016

[21] Yao J Gong X Shi X Fan S Chen J Chen Q. The efficacy of angiotensin converting enzyme inhibitors versus angiotensin II receptor blockers on insulin resistance in hypertensive patients: A protocol for a systematic review and meta-analysis. Medicine. (2020) 99:e20674. 10.1097/MD.0000000000020674 32541513 PMC7302663

[22] Nickenig G. Should angiotensin II receptor blockers and statins be combined? Circulation. (2004) 110:1013–20. 10.1161/01.CIR.0000139857.85424.45 15326080

[23] Scicchitano P Frasso G Carbone M Moncelli M Carbonara R Silvestris F. Late age at menarche increased common carotid artery intima-media thickness in overweight and obese women. J Otolaryngol Adv. (2013) 1:1–54. 10.14302/issn.2329-9487.jhc-12-154

[24] Tropeano AI Saleh N Hawajri N Macquin-Mavier I Maison P. Do all antihypertensive drugs improve carotid intima-media thickness? A network meta-analysis of randomized controlled trials. Fundam Clin Pharmacol. (2011) 25:395–404. 10.1111/j.1472-8206.2010.00832.x 20584209

[25] Yeh J-J Lin C-L Hsu N-H Kao C-H. Effects of statins and steroids on coronary artery disease and stroke in patients with interstitial lung disease and pulmonary fibrosis: a general population study. PLoS ONE. (2021) 16:e0259153. 10.1371/journal.pone.0259153 34705851 PMC8550436

[26] Yeh J-J Wei Y-F Lin C-L Hsu W-H. Association of asthma–chronic obstructive pulmonary disease overlap syndrome with coronary artery disease, cardiac dysrhythmia and heart failure: a population-based retrospective cohort study. BMJ Open. (2017) 7:e017657. 10.1136/bmjopen-2017-017657 28982831 PMC5640024

[27] Su VY Yang YH Perng DW Tsai YH Chou KT Su KC. Real-world effectiveness of medications on survival in patients with COPD-heart failure overlap. Aging. (2019) 11:3650–67. 10.18632/aging.102004 31175265 PMC6594806

[28] Chiang CE Wang TD Li YH Lin TH Chien KL Yeh HI. 2010 guidelines of the Taiwan Society of Cardiology for the management of hypertension. J Formos Med Assoc. (2010) 109:740–73. 10.1016/S0929-6646(10)60120-9 20970072

[29] Lin C-S Chu Y-H Hung Y-J Lee D-Y Chen C-Y. Outpatient hypertension control and prescribing habits for hypertension in Taiwan. Acta Cardiol Sin. (2013) 29oi:539–49. 27122755 PMC4805033

[30] Diabetes Association of the Republic of C. Executive summary of the DAROC clinical practice guidelines for diabetes care- 2018. J Formos Med Assoc. (2020) 119:577–86. 10.1016/j.jfma.2019.02.016 30952480

[31] Li YH Ueng KC Jeng JS Charng MJ Lin TH Chien KL. 2017 Taiwan lipid guidelines for high risk patients. J Formos Med Assoc. (2017) 116:217–48. 10.1016/j.jfma.2016.11.013 28242176

[32] Tseng L-N Tseng Y-H Jiang Y-D Chang C-H Chung C-H Lin BJ. Prevalence of hypertension and dyslipidemia and their associations with micro- and macrovascular diseases in patients with diabetes in Taiwan: an analysis of nationwide data for 2000–2009. J Formos Med Assoc. (2012) 111:625–36. 10.1016/j.jfma.2012.09.010 23217598

[33] Chou CW Kung PT Chou WY Tsai WC. Pay-for-performance programmes reduce stroke risks in patients with type 2 diabetes: a national cohort study. BMJ Open. (2019) 9:e026626. 10.1136/bmjopen-2018-026626 31619415 PMC6797306

[34] Jan C-F Chang C-J Hwang S-J Chen Y-C Yang H-Y Chen Y-C. Impact of team-based community healthcare on preventable hospitalisation: a population-based cohort study in Taiwan. BMJ Open. (2021) 11:e039986. 10.1136/bmjopen-2020-039986 33593765 PMC7888366

[35] Wang C-Y Tu S-T Sheu W Chen IC Chuang L-M Wu M-S. National survey of ABC (A1C, blood pressure, cholesterol) of Diabetes Health Promotion Institutes in Taiwan: 2002–2018. J Formos Med Assoc. (2018) 117:952–4. 10.1016/j.jfma.2018.08.013 30181014

[36] Scicchitano P Cameli M Maiello M Modesti PA Muiesan ML Novo S. Nutraceuticals and dyslipidaemia: beyond the common therapeutics. J Funct Foods. (2014) 6:11–32. 10.1016/j.jff.2013.12.006

[37] Cicero AFG Colletti A Bajraktari G Descamps O Djuric DM Ezhov M. Lipid lowering nutraceuticals in clinical practice: position paper from an International Lipid Expert Panel. Arch Med Sci. (2017) 13:965–1005. 10.5114/aoms.2017.69326 28883839 PMC5575230

[38] Cicero AFG Fogacci F Stoian AP Vrablik M Al Rasadi K Banach M. Nutraceuticals in the management of dyslipidemia: which, when, and for whom? Could nutraceuticals help low-risk individuals with non-optimal lipid levels? Curr Atheroscler Rep. (2021) 23:57. 10.1007/s11883-021-00955-y 34345932 PMC8332568

[39] Gliemann L. Dodging physical activity and healthy diet: can resveratrol take the edge off the consequences of your lifestyle? Am J Clin Nutr. (2020) 112:905–6. 10.1093/ajcn/nqaa199 32692802

[40] Ciccone M Scicchitano P Cortese F Gesualdo M Fornarelli F. Endothelial function in obese and overweight patients: the role of olive oil, fish and nuts. Int J Diabetes Clin Res. (2014) 2014:1. 10.23937/2377-3634/1410004

[41] Mattioli AV Palmiero P Manfrini O Puddu PE Nodari S Dei Cas A. Mediterranean diet impact on cardiovascular diseases: a narrative review. J Cardiovasc Med. (2017) 18:925–35. 10.2459/JCM.0000000000000573 28914660

[42] Hsieh HM Shin SJ Tsai SL Chiu HC. Effectiveness of pay-for-performance incentive designs on diabetes care. Med Care. (2016) 54:1063–9. 10.1097/MLR.0000000000000609 27479599

[43] Lee IT Hsu C-C Sheu WH-H Su S-L Wu Y-L Lin S-Y. Pay-for-performance for shared care of diabetes in Taiwan. J Formos Med Assoc. (2019) 118:S122–9. 10.1016/j.jfma.2019.08.011 31471222

[44] Balon R Rafanelli C Sonino N. Benzodiazepines: a valuable tool in the management of cardiovascular conditions. Psychother Psychosom. (2018) 87:327–30. 10.1159/000493015 30189429

[45] Bader DA Omer A El-Oedemi M. Anti-inflammatory effects of diazepam on different models of inflammation: roles ofperipheral benzodiazepine receptors and genes for corticosterone, nitric oxide andcytokines biosynthesis. J Clin Epigenet. (2017) 3:2. 10.21767/2472-1158.100053

[46] Huang W-S Muo C-H Chang S-N Chang Y-J Tsai C-H Kao C-H. Benzodiazepine use and risk of stroke: a retrospective population-based cohort study. Psychiatry Clin Neurosci. (2014) 68:255–62. 10.1111/pcn.12117 24829937

[47] Dominguini D Steckert AV Michels M Borges MS Ritter C Barichello T. The effects of anaesthetics and sedatives on brain inflammation. Neurosci Biobehav Rev. (2021) 127:504–13. 10.1016/j.neubiorev.2021.05.009 33992694

[48] Patorno E Glynn RJ Levin R Lee MP Huybrechts KF. Benzodiazepines and risk of all cause mortality in adults: cohort study. BMJ. (2017) 358:j2941. 10.1136/bmj.j2941 28684397 PMC5499256

[49] Cosci F Chouinard G. Acute and persistent withdrawal syndromes following discontinuation of psychotropic medications. Psychother Psychosom. (2020) 89:283–306. 10.1159/000506868 32259826

[50] Wu CK Huang YT Lee JK Jimmy Juang JM Tsai CT Lai LP. Anti-anxiety drugs use and cardiovascular outcomes in patients with myocardial infarction: a national wide assessment. Atherosclerosis. (2014) 235:496–502. 10.1016/j.atherosclerosis.2014.05.918 24953489

[51] Elkind MSV Boehme AK Smith CJ Meisel A Buckwalter MS. Infection as a stroke risk factor and determinant of outcome after stroke. Stroke. (2020) 51:3156–68. 10.1161/STROKEAHA.120.030429 32897811 PMC7530056

[52] Yao TC Huang YW Chang SM Tsai SY Wu AC Tsai HJ. Association between oral corticosteroid bursts and severe adverse events : a nationwide population-based cohort study. Ann Intern Med. (2020) 173:325–30. 10.7326/M20-0432 32628532

[53] Wang MT Liou JT Lin CW Tsai CL Wang YH Hsu YJ. Association of cardiovascular risk with inhaled long-acting bronchodilators in patients with chronic obstructive pulmonary disease: a nested case-control study. JAMA Intern Med. (2018) 178:229–38. 10.1001/jamainternmed.2017.7720 29297057 PMC5838614

[54] Waljee AK Rogers MAM Lin P Singal AG Stein JD Marks RM. Short term use of oral corticosteroids and related harms among adults in the United States: population based cohort study. BMJ. (2017) 357:j1415. 10.1136/bmj.j1415 28404617 PMC6284230

[55] Koarai A Ichinose M. Possible involvement of acetylcholine-mediated inflammation in airway diseases. Allergol Int. (2018) 67:460–6. 10.1016/j.alit.2018.02.008 29605098

[56] Keränen T Hömmö T Hämäläinen M Moilanen E Korhonen R. Anti-inflammatory effects of β2-receptor agonists salbutamol and terbutaline are mediated by MKP-1. PLoS ONE. (2016) 11:e0148144. 10.1371/journal.pone.0148144 26849227 PMC4743993

[57] Chen S Qiu A Tao Z Zhang H. Clinical impact of cardiovascular disease on patients with bronchiectasis. BMC Pulm Med. (2020) 20:101. 10.1186/s12890-020-1137-7 32326931 PMC7181495

[58] Yang Y-L Leu H-B Yin W-H Tseng W-K Wu Y-W Lin T-H. Adherence to healthy lifestyle improved clinical outcomes in coronary artery disease patients after coronary intervention. J Chin Med Assoc. (2021) 84:596–605. 10.1097/JCMA.0000000000000536 33871387 PMC12966064

[59] Wang CY Lai CC Wang YH Wang HC. The prevalence and outcome of short-acting β2-agonists overuse in asthma patients in Taiwan. NPJ Prim Care Respir Med. (2021) 31:19. 10.1038/s41533-021-00231-1 33879785 PMC8058069

[60] Su VY Yang KY Yang YH Tsai YH Perng DW Su WJ. Use of ICS/LABA combinations or LAMA is associated with a lower risk of acute exacerbation in patients with coexistent COPD and asthma. J Allergy Clin Immunol Pract. (2018) 6:1927–35.e1923. 10.1016/j.jaip.2018.01.035 29432960

[61] Chen Y-F Cheng Y-C Chou C-H Chen C-Y Yu C-J. Major comorbidities lead to the risk of adverse cardiovascular events in chronic obstructive pulmonary disease patients using inhaled long-acting bronchodilators: a case-control study. BMC Pulm Med. (2019) 19:233. 10.1186/s12890-019-0999-z 31795986 PMC6889444

[62] Samp JC Joo MJ Schumock GT Calip GS Pickard AS Lee TA. Risk of cardiovascular and cerebrovascular events in copd patients treated with long-acting β2-agonist combined with a long-acting muscarinic or inhaled corticosteroid. Ann Pharmacother. (2017) 51:945–53. 10.1177/1060028017719716 28677404

[63] Ish P Malhotra N Gupta N. GINA 2020: what's new and why? J Asthma. (2020) 58:1273–7. 10.1080/02770903.2020.1788076 32586146

[64] Flume PA Chalmers JD Olivier KN. Advances in bronchiectasis: endotyping, genetics, microbiome, and disease heterogeneity. Lancet. (2018) 392:880–90. 10.1016/S0140-6736(18)31767-7 30215383 PMC6173801

[65] Lujan M Gallardo X Amengual MJ Bosque M Mirapeix RM Domingo C. Prevalence of bronchiectasis in asthma according to oral steroid requirement: influence of immunoglobulin levels. Biomed Res Int. (2013) 2013:109219. 10.1155/2013/109219 24324951 PMC3845843

[66] Cheng C-L Lee C-H Chen P-S Li Y-H Lin S-J Yang Y-HK. Validation of acute myocardial infarction cases in the national health insurance research database in taiwan. J Epidemiol. (2014) 24:500–7. 10.2188/jea.JE20140076 25174915 PMC4213225

[67] Hsieh C-Y Su C-C Shao S-C Sung S-F Lin S-J Kao Yang Y-H. Taiwan's National Health Insurance Research Database: past and future. Clin Epidemiol. (2019) 11:349–58. 10.2147/CLEP.S196293 31118821 PMC6509937

[68] Yeh J-J Lin C-L Hsu C-Y Shae Z Kao C-H. Statin for tuberculosis and pneumonia in patients with asthma–chronic pulmonary disease overlap syndrome: a time-dependent population-based cohort study. J Clin Med. (2018) 7:381. 10.3390/jcm7110381 30355982 PMC6262333

[69] Huang HY Chung FT Lo CY Lin HC Huang YT Yeh CH. Etiology and characteristics of patients with bronchiectasis in Taiwan: a cohort study from 2002 to 2016. BMC Pulm Med. (2020) 20:45. 10.1186/s12890-020-1080-7 32070324 PMC7029505

[70] Hajian Tilaki K. Methodological issues of confounding in analytical epidemiologic studies. Caspian J Intern Med. (2012) 3:488–95. 24009920 PMC3755849

