# Supplementary material for: Relationships Between Bronchodilators, Steroids, Antiarrhythmic Drugs, Antidepressants, and Benzodiazepines and Heart Disease and Ischemic Stroke in Patients With Predominant Bronchiectasis and Asthma
Source: Front Cardiovasc Med. 2022 Feb 17;9:797623. doi: 10.3389/fcvm.2022.797623 (PMC8893278; doi:10.3389/fcvm.2022.797623)
Supplement: Supplementary file 1 [file Table_1.DOCX]

| MAJOR OUTCOME | | |
| --- | --- | --- |
|  | **ICD-9CM** | **FULL NAME** |
|  | 410 | acute myocardial infarction |
|  | 411 | other acute and subacute forms of ischemic heart disease, |
|  | 412 | old myocardial infarction, |
|  | 413 | angina pectoris, |
|  | 414 | other forms of chronic ischemic disease |
|  | 425 | cardiomyopathy , |
|  | 426 | conduction disorder, |
|  | 427 | cardiac arrhythmia |
|  | 428 | heart failure |
|  | 433 | Occlusion and stenosis of precerebral arteries |
|  | 434 | Occlusion of cerebral arteries |
|  | 435 | Transient cerebral ischemia |
|  | 436 | Acute but ill-defined cerebrovascular disease |
| COMOBORDITIES | | |
|  | 010-012, 480-486 | PTB  pulmonary tuberculosis |
|  | 0.31 | NTM  nontuberculous mycobacteria |
|  | 714.0 714.30 714.33 | rheumatoid arthritis |
|  | 710 | diffuse connective disease and Sjogren’s syndrome |
|  | 481, 482, 483, 485, and 486 | pneumonia |
|  | 491, 492, and 496 | chronic obstruction pulmonary disease |
|  | 250 and A181 | diabetes |
|  | 117.3 | aspergillosis |
|  | 112.4 and 112.5 | candiasis |
|  | 114.0, 114.3, 114.4, and 114.5 | endemic mycoses |
|  | 748.3 | Mounier-Kuhn |
|  | 277.0 | cystic fibrosis |
|  | 401-405, A260, and A269 | hypertension |
|  | 272.0-272.4 | hyperlipidemia |
|  | 415.1 | pulmonary embolism |
|  | 311 | depression |
|  | 305.1, 305.11, 305.12, and 305.13 | tobacco dependence |
|  | 649.01 | tobacco use disorder complicating pregnancy |
| DRUGS (MEDICATIONS) | | |
|  | **ATC** | **FULL NAMES** |
| BRONCHODILAORS | R03AC12  R03AC13 | LABAs  long-acting beta2 agonists  [Salmeterol](https://en.wikipedia.org/wiki/Salmeterol)  [Formotero](https://en.wikipedia.org/wiki/Formoterol)l |
|  | R03BB04 | LAMAs  long-acting muscarinic antagonists  [Tiotropium bromide](https://en.wikipedia.org/wiki/Tiotropium_bromide) |
|  | R03AC02,  R03AC03  R03AC04 | SABAs  short-acting beta2 agonists  [Salbutamol](https://en.wikipedia.org/wiki/Salbutamol)  [Terbutaline](https://en.wikipedia.org/wiki/Terbutaline)  [Fenoterol](https://en.wikipedia.org/wiki/Fenoterol) |
|  | R03BB01 | SAMAs  short-acting muscarinic antagonists  [Ipratropium bromide](https://en.wikipedia.org/wiki/Ipratropium_bromide) |
| STEROIDS | R03BA01, R03BA02,  R03BA05,  R03BA08 | ICSs  inhaled corticosteroids  [Beclometasone](https://en.wikipedia.org/wiki/Beclometasone)  [Budesonide](https://en.wikipedia.org/wiki/Budesonide)  Fluticasone  [Ciclesonide](https://en.wikipedia.org/wiki/Ciclesonide) |
|  | D07AC15  D07AC17 H02AB02 H02AB04 H02AB06  H02AB08  R01AD05  S01BA02 | OSs  oral steroids  [Beclometasone](https://en.wikipedia.org/wiki/Beclometasone)  [Fluticasone](https://en.wikipedia.org/wiki/Fluticasone_propionate)  [Dexamethasone](https://en.wikipedia.org/wiki/Dexamethasone)  [Methylprednisolone](https://en.wikipedia.org/wiki/Methylprednisolone)  [Prednisolone](https://en.wikipedia.org/wiki/Prednisolone)  [Triamcinolone](https://en.wikipedia.org/wiki/Triamcinolone)   \| Budesonide \|  \| \| --- \| --- \|   Hydrocortison |
| ANTI-ARRHYTHMIC | C01B | Anti-arrhythmic drugs |
| ANTIDEPRESSANTS | N06AB03 | Fluoxetine |
| BENZODIAZEPINES (BZDs) | N05BA12 | Alprazolam |
|  | N05BA17 | Fludiazepam |
| Statins C10AA | | |
| Antihypertension | | |
| Diuretics C03 | | |
| Beta blockers C07AB03 Atenol  (Cardioselective) C07AB07 Bisoprolol  C07AB02 Metoprolol | | |
| Angiotensin converting C09AA  enzyme inhibitors | | |
| Angiotensin-2 receptors C09CA  blockers | | |
| Calcium channel C08  blockers | | |
| PROCEDURES | | |
| CEST-X-RAY | 32001C 32002C |  |
| CT-RELATED | 33070B 33071B 33072B 33103B |  |
| PULMONARY FUNCTION-RELATED TEST | 17001C 17002B 17003C , 17006C, 17004B-17018B , 17019C ,  17020B - 17021B |  |
| ASTHMA-RELATED TEST and EXAMINATIONS | 12031C , 30021C-30022C , 30005B-30006B , 30009B-30010B , 30023B-30024B |  |
| CHEST REHABILITATION and ANTIBIOTIC | | |
| PHYSIOTHERAPY | 42001～42019 | Pre & post chest operation  Bronchiectasis Asthma |
| ANTIOBITICS | J01A  J01B  J01C  J01D  J01E  J01F  J01G  J01M  J01X | Tetracyclines  Amphenicols  Penicillins  Other beta-lacta  Cephalosporins  Macrolides  Aminoglycosides  Quinolones  OTHER |
